# Early Prediction of Volumetric Progression in Sellar–Parasellar Meningiomas Using Delta Radiomics on 6-Month MRI Following Gamma Knife Radiosurgery

**DOI:** 10.3390/diagnostics16152473

**Published:** 2026-08-05

**Authors:** Merve Yazol, Halil Özer, Pelin Kuzucu, Burak Karaaslan

**Affiliations:** 1 Department of Radiology, Faculty of Medicine, Gazi University, Ankara 06500, Turkey; 2 Department of Radiology, Faculty of Medicine, Selcuk University, Konya 42130, Turkey; drhalilozer@gmail.com; 3 Department of Neurosurgery, Faculty of Medicine, Gazi University, Ankara 06500, Turkey; drpelinkuzucu@gmail.com (P.K.); burakkaraaslan08@hotmail.com (B.K.)

**Keywords:** Gamma Knife radiosurgery, radiomics, delta radiomics, apparent diffusion coefficient, meningioma, machine learning

## Abstract

**Background/Objectives**: This study aimed to develop and internally validate multiparametric MRI radiomics models for predicting volumetric progression following Gamma Knife radiosurgery (GKRS) in sellar–parasellar meningiomas and to evaluate the incremental value of diffusion-derived features beyond contrast-enhanced imaging. **Methods**: Fifty-four patients underwent pretreatment and approximately 6-month post-treatment MRI, including contrast-enhanced T1-weighted imaging (T1C-WI) and apparent diffusion coefficient (ADC) maps. Whole-tumor segmentations were reviewed by two neuroradiologists by consensus. Radiomic features were extracted using PyRadiomics, and delta features were calculated as post-treatment minus pretreatment values. Elastic-net logistic regression models were evaluated using repeated nested cross-validation with five-fold inner and outer loops repeated 10 times. The primary endpoint was volumetric progression, defined as a >20% volume increase at 3 years. **Results**: At 3 years, 9 tumors (16.7%) progressed, 25 (46.3%) remained stable, and 20 (37.0%) regressed. The ΔT1C-WI model showed the highest repeated nested cross-validation performance, with a mean AUC of 0.861 ± 0.065, an accuracy of 0.846 ± 0.037, and an F1 score of 0.579 ± 0.097. Averaged patient-level out-of-fold predictions yielded an AUC of 0.914 (95% CI, 0.822–0.980), a sensitivity of 77.8%, and a specificity of 88.9%. The ΔADC model showed moderate discrimination, whereas combining ΔT1C-WI and ΔADC features did not improve performance. **Conclusions**: Delta radiomics derived from 6-month post-treatment T1C-WI may help identify sellar–parasellar meningiomas at risk of 3-year volumetric progression after GKRS. These findings suggest that 6-month ΔT1C-WI radiomics may support early risk stratification, but its clinical value requires external validation and prospective evaluation.

## 1. Introduction

Sellar and parasellar meningiomas present significant diagnostic and therapeutic challenges due to their proximity to critical neurovascular structures such as the optic chiasm and cavernous sinus [1]. Gamma Knife radiosurgery (GKRS) is a well-established, noninvasive treatment option, particularly for residual, recurrent, or surgically inaccessible tumors [1,2]. Although long-term tumor control rates are favorable, a subset of lesions shows delayed volumetric progression; reliable early-response biomarkers are therefore needed.

Conventional MRI assessment primarily relies on tumor size changes, which may be insensitive to early microstructural alterations in indolent meningiomas, particularly when volumetric response is delayed beyond 12–18 months after treatment [3,4]. Transient volume increases related to perilesional edema occur in up to 40% of patients within the first year after GKRS, and this complicates response assessment based solely on size criteria [5]. This phenomenon, termed pseudoprogression, can be misinterpreted as treatment failure despite eventual tumor control, potentially leading to unnecessary interventions.

Diffusion-weighted imaging (DWI) and apparent diffusion coefficient (ADC) maps allow assessment of microstructural alterations associated with tumor response [6,7]. Although mean ADC values have been used as markers of treatment response, single-parameter measurements do not adequately capture intratumoral spatial heterogeneity. Radiomics provides a more comprehensive framework by extracting quantitative imaging features that reflect tumor biology, including first-order statistics, shape descriptors, and higher-order texture patterns [8]. In gliomas and brain metastases, radiomic features derived from structural and diffusion MRI have demonstrated improved predictive performance compared with conventional imaging metrics [9,10,11]. In meningiomas, baseline radiomics derived from contrast-enhanced T1-weighted imaging (T1C-WI) has been associated with WHO grade, Ki-67 index, and recurrence risk [3,9]. However, these approaches are inherently static and do not account for treatment-induced biological adaptation, particularly following radiosurgery.

Machine learning (ML) approaches enable the integration of multiparametric radiomic features and have shown promising results in neuro-oncology [6,12,13]. Longitudinal radiomics, or “delta radiomics,” quantifies feature changes across imaging timepoints and may capture treatment-related changes that are not reflected in baseline imaging. However, direct comparisons between baseline radiomics and delta radiomics remain limited, particularly in the context of radiosurgical treatment [14]. The predictive value of multiparametric MRI-derived delta radiomics for forecasting long-term progression after GKRS in sellar–parasellar meningiomas remains insufficiently explored. In particular, the value of longitudinal changes between pretreatment and early post-treatment imaging, as well as the additional contribution of diffusion-derived radiomic features, remains unclear.

Therefore, this study aimed to develop and internally validate multiparametric MRI radiomics models combined with machine learning to predict volumetric progression at 3 years following GKRS in sellar–parasellar meningiomas using delta radiomic features derived from 6-month post-treatment imaging. We also evaluated the added value of diffusion-derived features beyond contrast-enhanced imaging.

## 2. Materials and Methods

### 2.1. Study Population

This retrospective single-center study included patients with sellar–parasellar meningiomas treated with GKRS. Patients were identified from the institutional radiosurgery database. The institutional review board approved the study and waived informed consent due to its retrospective design (2026-425).

Inclusion criteria were (1) histologically confirmed or radiologically presumed sellar or parasellar meningioma; (2) treatment with single-session GKRS; (3) availability of pretreatment MRI within one month before GKRS and follow-up MRI at approximately 6 months, including T1C-WI and DWI with corresponding ADC maps; (4) availability of T1C-WI at approximately 3 years after GKRS for volumetric outcome assessment; and (5) adequate image quality without significant motion or susceptibility artifacts. Patients were excluded if they had inadequate MRI quality, missing required MRI sequences, insufficient follow-up, prior radiotherapy or radiosurgery, or additional intracranial pathology that could confound imaging analysis.

The 6-month MRI was the first routinely scheduled post-GKRS examination and the earliest timepoint at which both T1C-WI and ADC maps were consistently available for all eligible patients.

Eligibility for the study was based on imaging and follow-up availability and was considered separately from the clinical indication for GKRS. Primary GKRS was defined as radiosurgery without prior tumor resection, whereas salvage GKRS was defined as radiosurgery after previous surgery for residual or recurrent disease. Selection for primary GKRS was based on multidisciplinary evaluation. The main considerations were unfavorable tumor location with high anticipated surgical morbidity, advanced age or medical comorbidity, patient preference, and, in selected cases, small tumor volume combined with unfavorable location.

**Figure 1:**
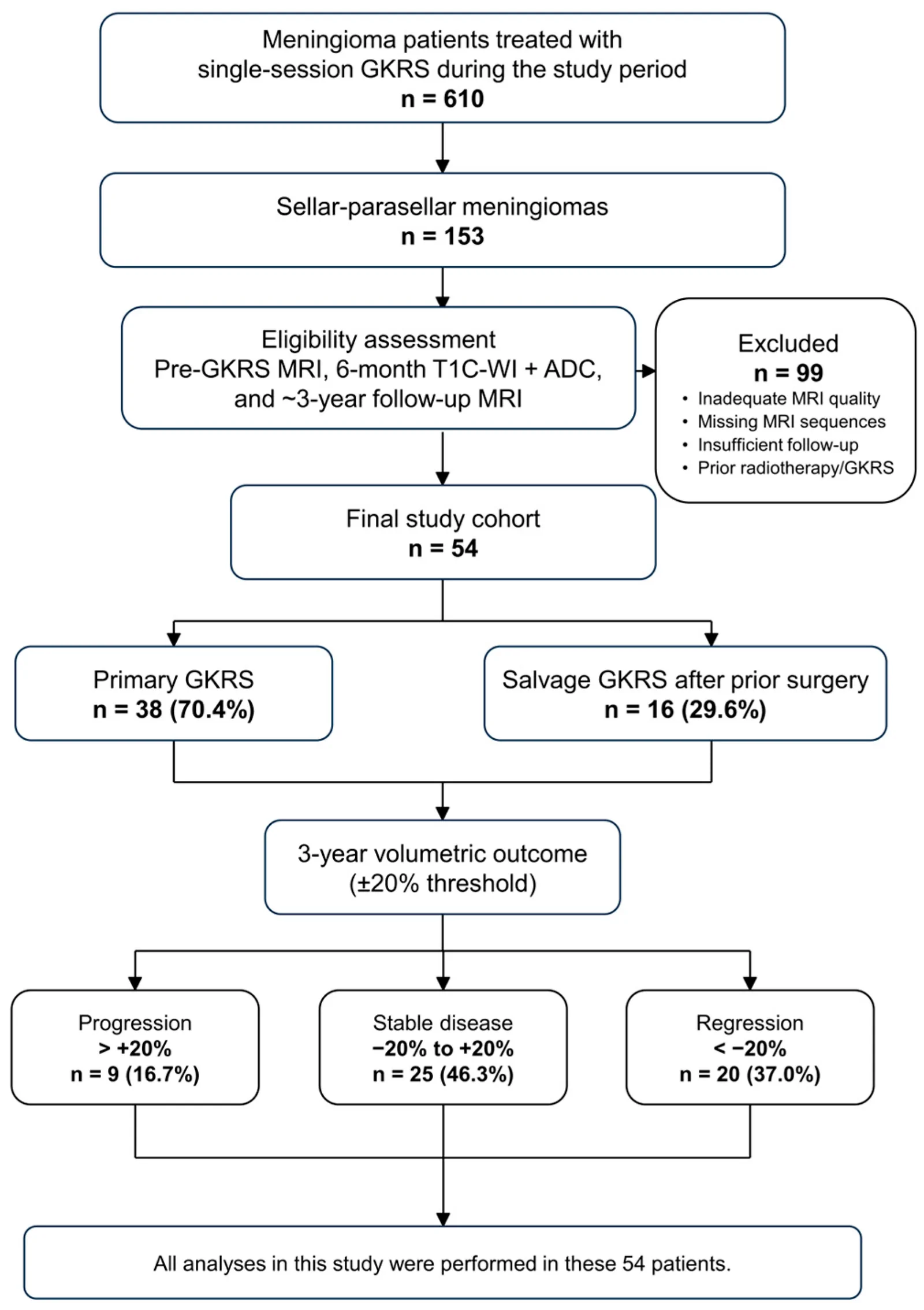
Patient-selection flowchart. Flowchart summarizing patient screening, exclusions, the final study cohort, treatment setting as primary or salvage GKRS, and 3-year volumetric outcome categories.

**Figure 2:**
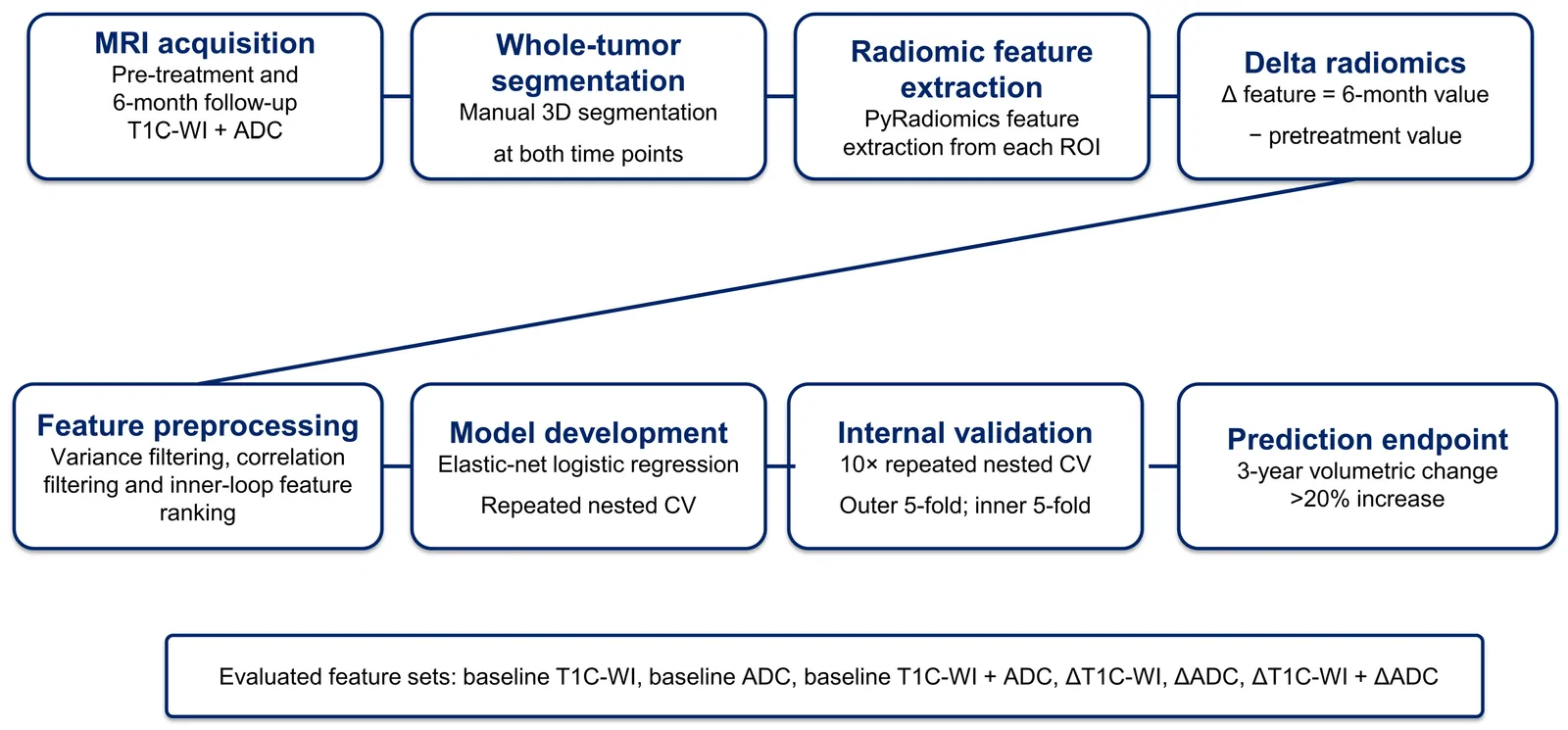
Study workflow. T1C-WI and ADC maps were segmented, and radiomic features were extracted; delta features (6-month minus pretreatment) were used in elastic-net models with nested cross-validation to predict 3-year volumetric progression defined as a >20% increase in tumor volume.

A total of 54 patients met the inclusion criteria. All consecutive eligible patients available during the study period were included; no formal a priori sample-size calculation was performed. The final cohort comprised 38 patients treated with primary GKRS and 16 treated with salvage GKRS after previous surgery. Patient selection and cohort classification are summarized in Figure 1, and the delta-radiomics workflow is shown in Figure 2.

### 2.2. Radiosurgery Treatment Protocol

During radiosurgery planning, tumor volumes and organs at risk were delineated by a neurosurgeon and a medical physicist using T1C-WI. All patients received a single prescription dose of 9–20 Gy, delivered with the Leksell Gamma Knife Perfexion system (Elekta AB, Stockholm, Sweden). The prescription dose was determined according to tumor size, location, and proximity to critical neurovascular structures.

### 2.3. Imaging Protocol

All MRI examinations were performed on a single 1.5T scanner (MAGNETOM Aera; Siemens Healthineers, Erlangen, Germany)). Radiomic analysis was based on pre-treatment MRI obtained within 4 weeks before GKRS and post-treatment MRI performed at approximately 6 months. T1C-WI obtained at approximately 3 years after GKRS was used for volumetric outcome assessment.

T1C-WI was acquired in the axial plane using a three-dimensional FLASH gradient-echo sequence after intravenous administration of a gadolinium-based contrast agent (TR/TE = 9.8/4.76 ms, flip angle = 25°, slice thickness = 1 mm, acquisition matrix = 256 × 256, field of view = 250 × 250 mm, voxel size = 1.0 × 1.0 × 1.0 mm). DWI was acquired before contrast administration in the axial plane using a RESOLVE readout-segmented echo-planar imaging sequence with b-values of 0 and 1000 s/mm^2^ (TR/TE = 5760/61 ms, slice thickness = 4 mm, spacing between slices = 5.2 mm, acquisition matrix = 164 × 164, field of view = 218 × 218 mm, in-plane pixel spacing = 1.72 × 1.72 mm, pixel bandwidth = 895 Hz/pixel). ADC maps were computed automatically on the scanner workstation using a monoexponentially model fitted to b = 0 and b = 1000 s/mm^2^ images. Only T1C-WI and ADC maps were included in the radiomic analysis, as these sequences were consistently acquired across all patients.

### 2.4. Tumor Segmentation

**Figure 3:**
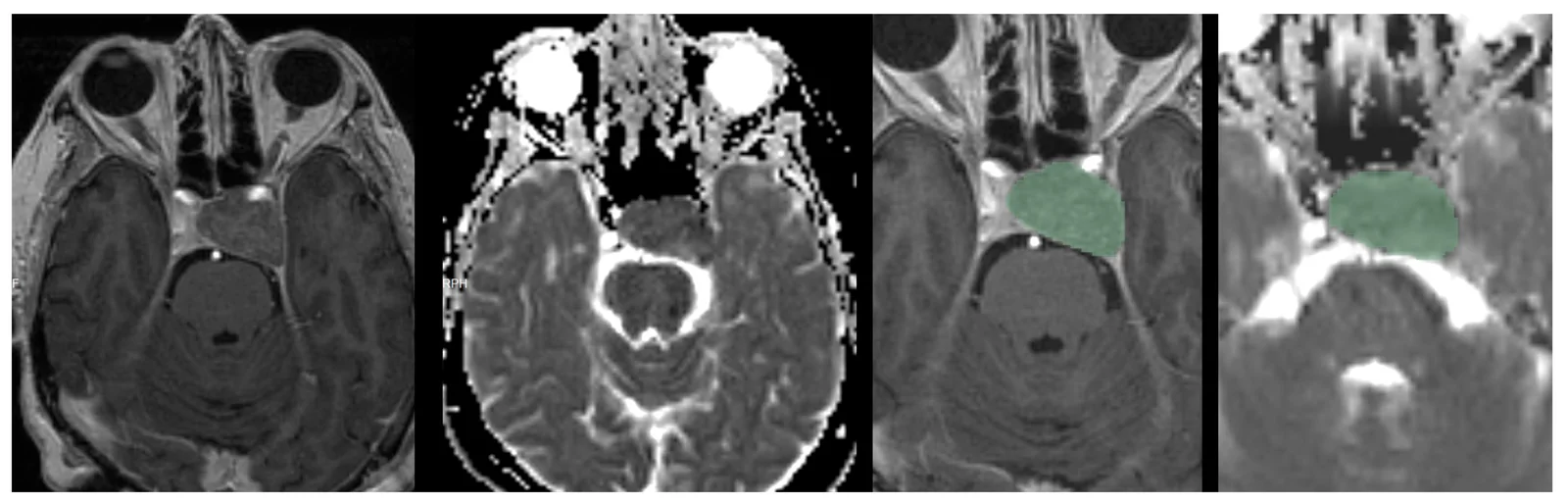
Representative segmentation example of a left cavernous sinus meningioma. Contrast-enhanced T1-weighted images and ADC maps are shown with the corresponding manually delineated whole-tumor segmentations.

Whole-tumor segmentation was performed manually, slice by slice, on pretreatment and approximately 6-month T1C-WI and ADC maps using the Segment Editor module in 3D Slicer (version 5.10.0) [15]. Each image set was segmented separately without sequence registration. Tumor boundaries were reviewed on every slice, and the final segmentations used for analysis were established by consensus between two neuroradiologists with more than 10 years of experience. A representative segmentation example is shown in Figure 3.

### 2.5. Segmentation Reproducibility

To assess robustness against inter-reader segmentation variability, a randomly selected subset of 15 patients, corresponding to 27.8% of the study cohort, was independently segmented by both neuroradiologists using the same segmentation protocol. Radiomic features were extracted from each segmentation using the same PyRadiomics workflow. Reproducibility of ΔT1C-WI radiomic features was assessed using an intraclass correlation coefficient based on a two-way random-effects model, absolute agreement, and single measurements [ICC(2,1)]. Features with ICC values ≥ 0.80 were considered reproducible. This analysis was performed as a supporting robustness assessment. Of the 107 evaluated ΔT1C-WI radiomic features, 47 met the ICC ≥ 0.80 threshold. Complete feature-level results are provided in Supplementary Table S1.

### 2.6. Treatment Response Assessment

Treatment response was assessed based on the volumetric change between pre-treatment and 3-year follow-up T1C-WI. Predictive models were developed using delta radiomics features calculated from pre-treatment and 6-month post-treatment MRI, allowing prediction of 3-year volumetric progression from 6-month imaging. The percentage change was calculated as:

Volume change (%) = (V_3_ᵧ − V_0_)/V_0_ × 100

Tumors were classified into three response groups: regression, defined as a volume decrease >20%; stable disease, defined as a volume change between −20% and +20%; and progression, defined as a volume increase >20%. These thresholds were selected in accordance with previously reported criteria for GKRS response assessment in meningiomas [4,16,17]. Tumor control was defined as regression or stable disease at 3-year follow-up. For the primary analysis, a binary classification task was defined in which tumors demonstrating progression were compared with non-progressive tumors, including both stable disease and regression. The distribution of volumetric response categories is summarized in Table 1.

As a secondary benchmark, bidimensional response was assessed using the imaging component of the RANO meningioma framework. The longest tumor diameter and its longest perpendicular diameter were measured on comparable contrast-enhanced T1-weighted imaging planes. Progressive disease was defined as a ≥25% increase in the sum of the products of perpendicular diameters relative to the nadir measurement [18]. This assessment did not replace the predefined three-dimensional volumetric endpoint.

### 2.7. Image Preprocessing and Radiomic Feature Extraction

Radiomic features were extracted from segmented tumor volumes on T1C-WI and ADC maps at both pre-treatment and 6-month post-treatment timepoints using the SlicerRadiomics extension of 3D Slicer (version 5.10.0), which implements the PyRadiomics framework [15]. Feature extraction was performed separately for each sequence and timepoint, yielding four feature sets per patient, and feature definitions and calculations were based on the Image Biomarker Standardisation Initiative framework [19].

T1C-WI images were resampled to an isotropic voxel size of 1.0 × 1.0 × 1.0 mm using B-spline interpolation, intensity-normalized with a normalization scale of 100, and discretized using a fixed bin width of 5. ADC maps were resampled to 2.0 × 2.0 × 2.0 mm using linear interpolation and discretized using a fixed bin width of 25. Intensity normalization was not applied to ADC maps to preserve their native quantitative values. N4 bias-field correction was not performed. Feature extraction was conducted in three dimensions using the original image type. Pre-cropping was enabled with a pad distance of 10 voxels, using label value 1 and a geometry tolerance of 0.001.

From each region of interest, 107 radiomic features were obtained, including shape (*n* = 14), first-order statistics (*n* = 18), and texture features derived from the gray-level co-occurrence matrix (GLCM, *n* = 24), gray-level run-length matrix (GLRLM, *n* = 16), gray-level size-zone matrix (GLSZM, *n* = 16), gray-level dependence matrix (GLDM, *n* = 14), and neighborhood gray tone difference matrix (NGTDM, *n* = 5). Wavelet and Laplacian-of-Gaussian filtered images were excluded to limit dimensionality and preserve interpretability. Volume-derived features (MeshVolume, VoxelVolume) were removed to avoid circularity with the volumetric outcome definition.

### 2.8. Delta Radiomics

To characterize treatment-related imaging alterations, delta radiomic features were calculated as the difference between 6-month post-treatment and pre-treatment feature values for each patient:

ΔFeature = Feature_6m_ − Feature_0_

Delta radiomic features were calculated separately for T1C-WI and ADC maps, generating two feature sets: ΔT1C-WI and ΔADC. A combined multiparametric feature set incorporating both ΔT1C-WI and ΔADC features was subsequently constructed to assess the incremental value of diffusion-derived features. Baseline radiomic features extracted from pretreatment images were also analyzed to determine whether pretreatment characteristics alone could predict outcome.

### 2.9. Machine Learning Pipeline

Predictive modeling was performed using an elastic-net-regularized logistic regression classifier implemented in Python with the scikit-learn library. Elastic-net regularization combines L1 and L2 penalties, enabling feature shrinkage and embedded variable selection in a high-dimensional radiomics setting. This approach was selected because the limited cohort, small number of progression events, and high-dimensional set of correlated radiomic features required a strongly regularized and interpretable modeling strategy.

To avoid information leakage, all preprocessing steps were embedded within the model-training pipeline and applied exclusively within the training folds of cross-validation. The pipeline consisted of median imputation for any missing values, variance threshold filtering to remove zero-variance features, z-score standardization, mutual information–based feature selection retaining up to 80 top-ranked features as an initial dimensionality-reduction step, and correlation filtering to eliminate highly redundant variables with an absolute Pearson correlation coefficient >0.90. Class imbalance was addressed using inverse class-frequency weighting within the elastic-net logistic regression classifier. Stratified folds were used in both the inner and outer cross-validation loops to preserve the progression/non-progression distribution during hyperparameter tuning and model evaluation. No synthetic oversampling or undersampling was applied.

### 2.10. Model Training and Validation

Model performance was evaluated using repeated nested cross-validation. The outer validation loop used stratified five-fold cross-validation, while the inner loop also used stratified five-fold cross-validation for hyperparameter optimization. This nested procedure was repeated 10 times using different random seeds. Within the inner loop, the elastic-net hyperparameters were optimized by grid search. Regularization strength (C) was evaluated at 0.1, 0.3, 1, 3, and 10, while the L1–L2 mixing parameter (l1_ratio) was evaluated at 0.1, 0.3, 0.5, 0.7, and 0.9. The SAGA solver was used for model optimization. All data-dependent preprocessing and feature-selection steps, including median imputation, variance filtering, standardization, mutual-information-based feature selection, and correlation filtering, were incorporated within the modeling pipeline and fitted using only the corresponding training data. After hyperparameter optimization in the inner loop, the optimized model was refitted on the complete outer training partition and evaluated in the held-out outer fold.

Repeated nested cross-validation was selected to reduce optimism arising from hyperparameter selection and model development in a limited dataset. Hyperparameters were optimized within the inner loop, whereas the outer loop was used exclusively to estimate model performance on unseen data. This separation reduced information leakage, optimistic performance estimation, and overfitting risk. Repeating this procedure with different random seeds reduced dependence on a single data partition and allowed variability in model performance across different data splits to be assessed. Given the limited number of progression events (n = 9), the findings should be interpreted as exploratory and require external validation.

For the primary binary progression model, ΔT1C-WI radiomics was used as the main feature set. Additional analyses were performed using baseline T1C-WI, baseline ADC, combined baseline T1C-WI + ADC, ΔADC, and combined ΔT1C-WI + ΔADC radiomics to assess the relative and incremental predictive value of each imaging input. Baseline tumor volume was additionally evaluated as a standalone secondary benchmark and in combination with ΔT1C-WI radiomics using the same repeated nested cross-validation framework.

### 2.11. Statistical Analysis and Performance Metrics

The mean area under the receiver operating characteristic curve (AUC) across repeated nested cross-validation runs was the primary performance metric; accuracy, F1 scores, and their standard deviations were also reported. Averaged patient-level out-of-fold probabilities were used to calculate the confusion matrix, sensitivity, specificity, accuracy, and Brier score. The AUC 95% confidence interval was estimated using 10,000 stratified patient-level bootstrap resamples, while exact binomial 95% confidence intervals were calculated for sensitivity, specificity, and accuracy at the prespecified threshold of 0.50. ROC curves from the same patients were compared using paired DeLong tests. Calibration was assessed using five quantile-based groups and the Brier score, and exploratory decision curve analysis compared the model with treat-all and treat-none strategies across threshold probabilities of 0.01–0.80.

Feature stability was defined as the proportion of the 50 outer-fold models in which each feature retained a non-zero coefficient. A full-cohort elastic-net refit was used only to construct an exploratory nomogram-style representation of the selected ΔT1C-WI features and was not used for performance estimation.

Available GKRS treatment parameters were summarized as the median and interquartile range and compared between the progression and non-progression groups using exploratory Mann–Whitney U tests. These parameters were not included in the radiomics models.

Reporting was aligned with the applicable TRIPOD + AI recommendations, while radiomics methodology and reporting were reviewed using the Radiomics Quality Score and CLEAR frameworks [20,21,22]. A completed CLEAR and TRIPOD + AI checklist are provided as Supplementary Checklists S1 and S2. Radiomic analysis used 3D Slicer 5.10.0 and PyRadiomics 3.1.0a2.post10; machine-learning and statistical analyses used Python 3.11.15, NumPy 2.4.6, pandas 3.0.3, SciPy 1.17.1, scikit-learn 1.8.0, and Matplotlib 3.10.9.

## 3. Results

### 3.1. Patient Characteristics and Volumetric Outcomes

Fifty-four patients with sellar–parasellar meningiomas treated with GKRS were included. The cohort comprised 41 women (75.9%) and 13 men (24.1%), with a mean age of 54.4 ± 11.8 years (range, 30–78 years). Thirty-eight patients underwent primary GKRS and 16 underwent salvage GKRS after surgery. Histopathological confirmation was available in all 16 previously operated patients. Progression occurred in 5 of 38 primary cases and 4 of 16 salvage cases, with no significant association between treatment setting and 3-year volumetric progression (Fisher’s exact test, *p* = 0.425). The median GKRS prescription dose was 13.0 Gy (interquartile range, 12.0–13.8 Gy; range, 9–20 Gy). Demographic, clinical, treatment, and volumetric characteristics are summarized in Table 1.

Among the 38 patients treated with primary GKRS, the documented indications were unfavorable tumor location or high anticipated surgical morbidity in 23 (60.5%), age or comorbidities in 8 (21.1%), patient preference against surgery in 6 (15.8%), and small tumor volume together with unfavorable location in 1 (2.6%). Thirty-two of the 38 primary GKRS tumors had a baseline volume below 10 cm^3^. Detailed indications are provided in Supplementary Table S2. Among the 16 patients treated with salvage GKRS, 12 had undergone one previous resection and four had undergone two. Fifteen had undergone subtotal resection and one gross-total resection. Simpson grade was recorded as grade IV in 14 patients and grade II in two. Thirteen patients had residual disease and three had recurrent disease. The median interval from the most recent surgery to GKRS was 5.2 months (range, 1.0–43.0 months). Detailed surgical and radiosurgical characteristics are provided in Supplementary Tables S3 and S4.

At 3-year follow-up, tumor progression was observed in 9 patients (16.7%), stable disease in 25 (46.3%), and regression in 20 (37.0%) according to the predefined ±20% volumetric change threshold, yielding a tumor control rate of 83.3%. For the primary binary analysis, the cohort comprised 9 progression and 45 non-progression cases.

The mean baseline tumor volume was 8.0 ± 9.6 cm^3^ (median, 5.2 cm^3^; range, 0.6–51.2 cm^3^), and the mean 3-year follow-up volume was 7.3 ± 9.0 cm^3^ (median, 4.9 cm^3^; range, 0.6–47.5 cm^3^). The mean absolute volume change was −0.73 ± 3.26 cm^3^, and the mean percentage volume change was −8.2 ± 26.3%. Progressive tumors showed a mean volume increase of 39.9 ± 16.5% (range, 26.1–76.7%), stable tumors showed a mean change of −6.9 ± 9.5% (range, −18.3% to 13.7%), and regressing tumors showed a mean decrease of 31.4 ± 6.5% (range, −48.0% to −20.6%).

Exploratory univariable analyses showed higher prescription and maximum doses and fewer shots in the progression group than in the non-progression group. Prescription isodose line and target volume did not differ significantly. Detailed results are provided in Supplementary Table S4.

Using the imaging component of the RANO meningioma framework as a secondary benchmark, 8 tumors were classified as progressive disease, 31 as stable disease, 12 as minor response, and 3 as partial response. The bidimensional and three-dimensional classifications agreed in 53 of 54 patients (98.1%; κ = 0.930). Eight of the 9 tumors classified as progressive by volumetric assessment also met the bidimensional RANO progression criterion. The remaining tumor showed a 14.3% increase in bidimensional product and was therefore classified as stable by the RANO benchmark. Detailed results are provided in Supplementary Table S5.

### 3.2. Segmentation Reproducibility Results

In the 15-patient interobserver segmentation subset, 47 of 107 ΔT1C-WI radiomic features achieved an ICC of at least 0.80. Complete feature-level reproducibility results are provided in Supplementary Table S1.

### 3.3. Comparative Performance of Binary Progression Models

Baseline radiomics models showed limited discrimination for 3-year volumetric progression. The baseline T1C-WI model achieved a mean AUC of 0.686 ± 0.117, accuracy of 0.757 ± 0.033, and F1 score of 0.392 ± 0.149. The baseline ADC model showed lower performance, with a mean AUC of 0.592 ± 0.102, accuracy of 0.669 ± 0.060, and F1 score of 0.294 ± 0.124. Combining baseline T1C-WI and ADC features yielded a mean AUC of 0.695 ± 0.109, accuracy of 0.765 ± 0.045, and F1 score of 0.415 ± 0.125.

**Figure 4:**
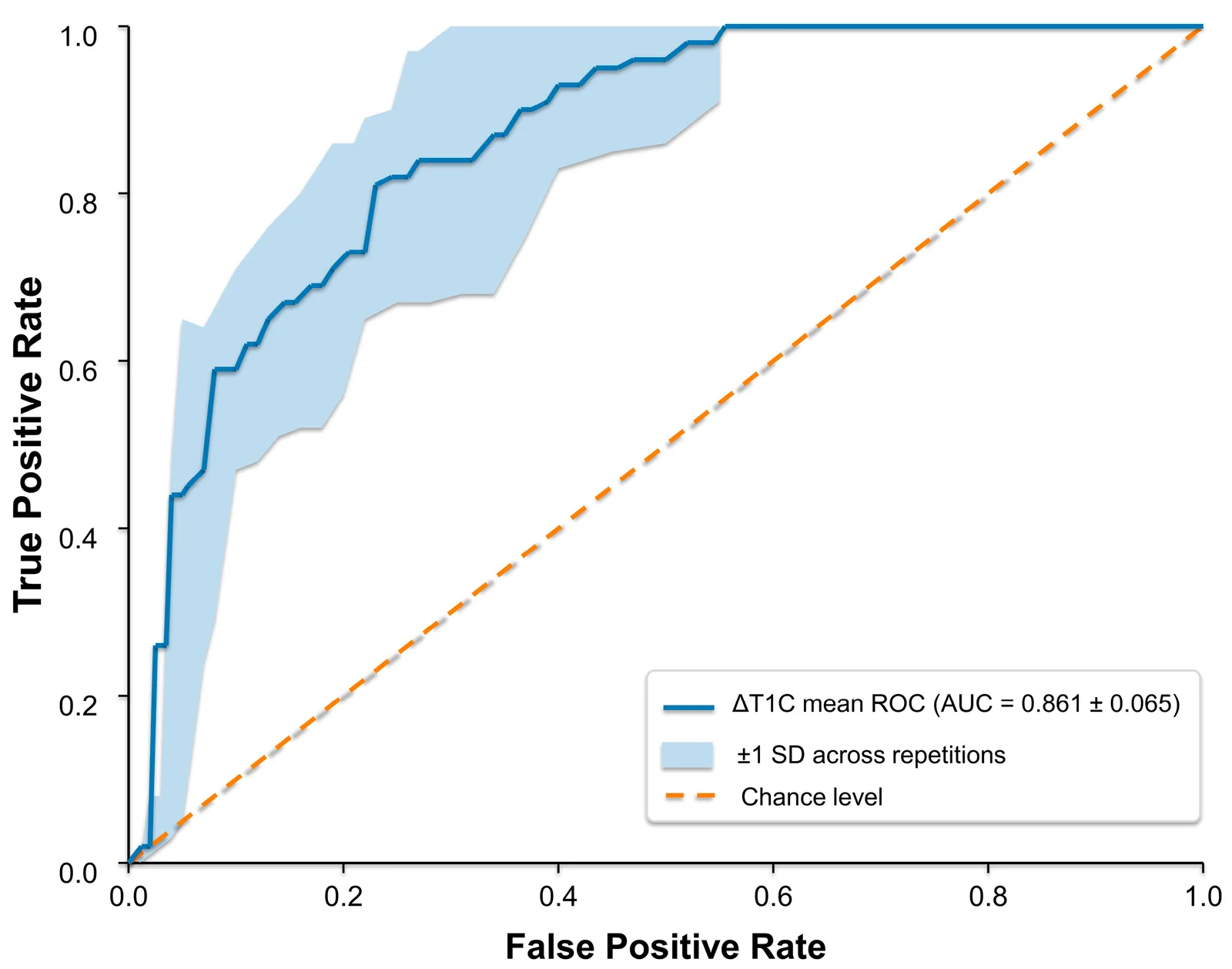
Mean receiver operating characteristic curve for the primary ΔT1C-WI progression model across repeated nested cross-validation runs. The mean AUC was 0.861 ± 0.065.

**Figure 5:**
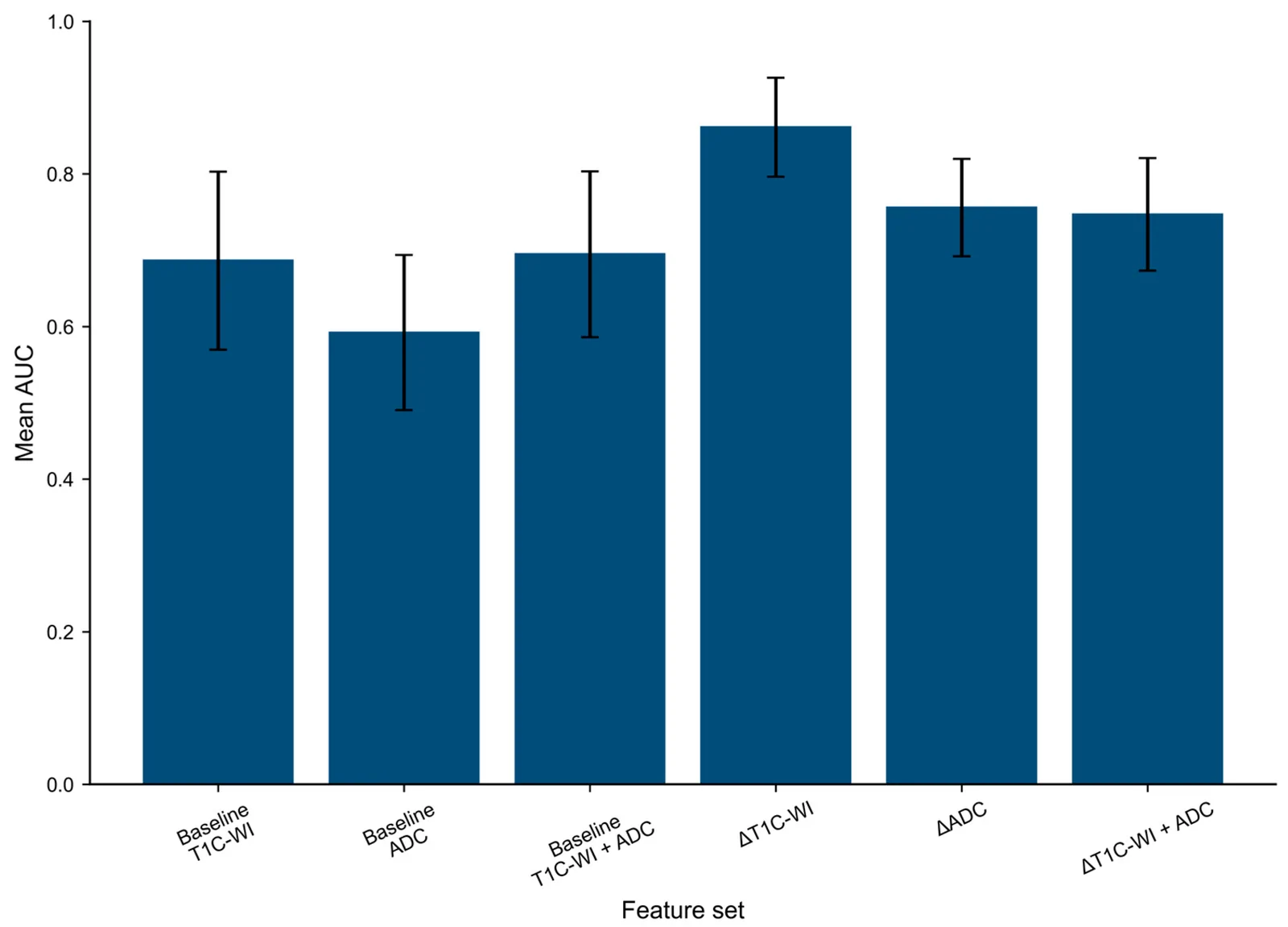
Comparison of binary prediction models. Mean AUCs from repeated nested cross-validation are shown for the primary ΔT1C-WI model and the secondary baseline, diffusion, and combined-radiomics models.

Models based on longitudinal radiomic changes showed higher predictive performance. The ΔT1C-WI model achieved the strongest discrimination, with a mean AUC of 0.861 ± 0.065, accuracy of 0.846 ± 0.037, and F1 score of 0.579 ± 0.097. The ΔADC model showed moderate predictive value, with a mean AUC of 0.756 ± 0.064, accuracy of 0.787 ± 0.046, and F1 score of 0.457 ± 0.091. Combining ΔT1C-WI and ΔADC features did not improve performance, yielding a mean AUC of 0.747 ± 0.074, accuracy of 0.802 ± 0.033, and F1 score of 0.426 ± 0.086. Model performance is summarized in Table 2, and comparative discrimination is shown in Figure 4 and Figure 5.

Baseline tumor volume alone showed limited discrimination (repeated nested-CV AUC, 0.607 ± 0.042), and its addition to the ΔT1C-WI model did not meaningfully improve performance compared with ΔT1C-WI alone (0.853 ± 0.085 vs. 0.861 ± 0.065).

The primary ΔT1C-WI model showed higher patient-level out-of-fold AUCs than the baseline T1C-WI, ΔADC, and combined ΔT1C-WI + ΔADC models; however, none of the paired DeLong comparisons reached statistical significance (*p* = 0.088, *p* = 0.128, and *p* = 0.144, respectively).

### 3.4. Primary ΔT1C-WI Progression Model

**Figure 6:**
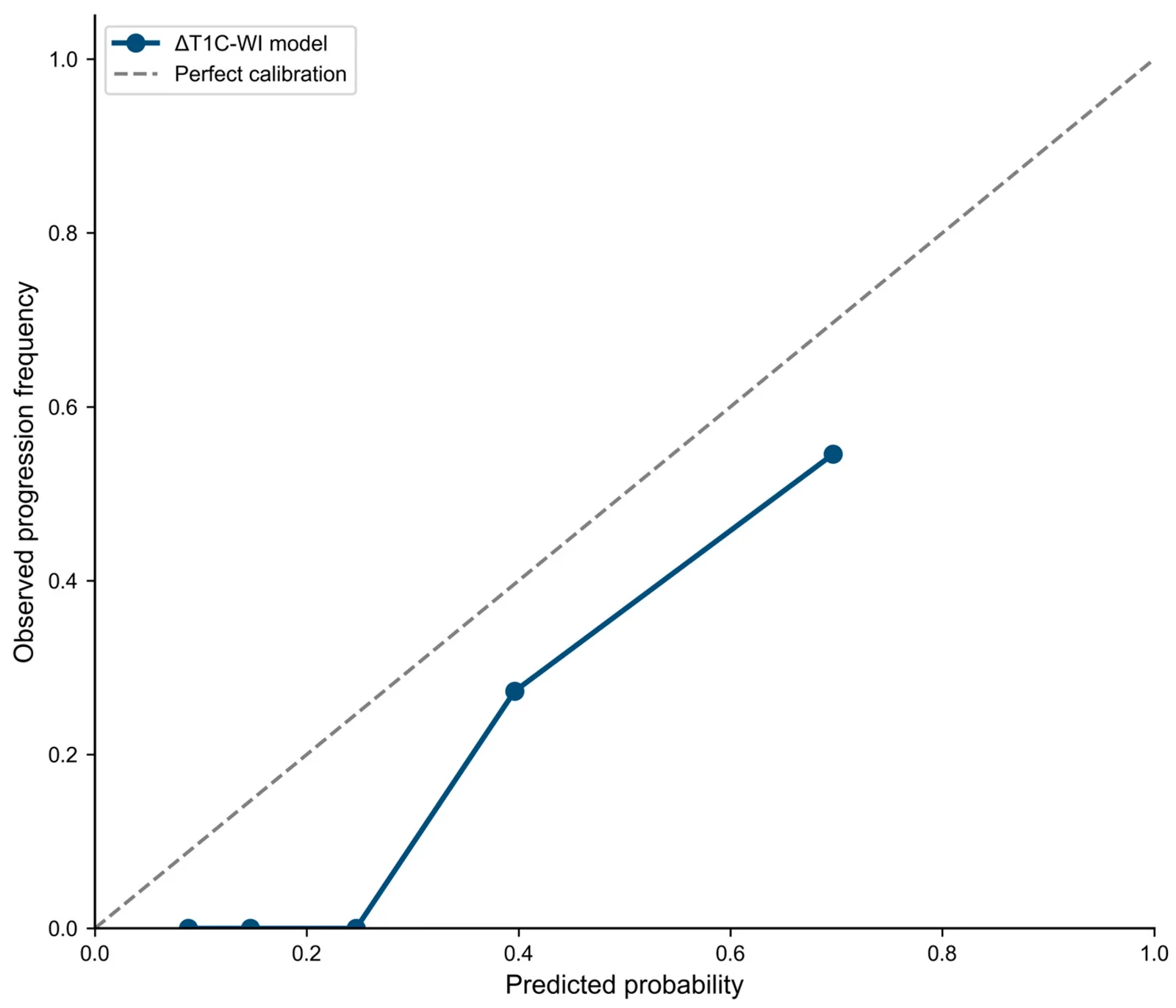
Calibration curve for the primary ΔT1C-WI progression model based on averaged patient-level out-of-fold probabilities. The Brier score was 0.112.

**Figure 7:**
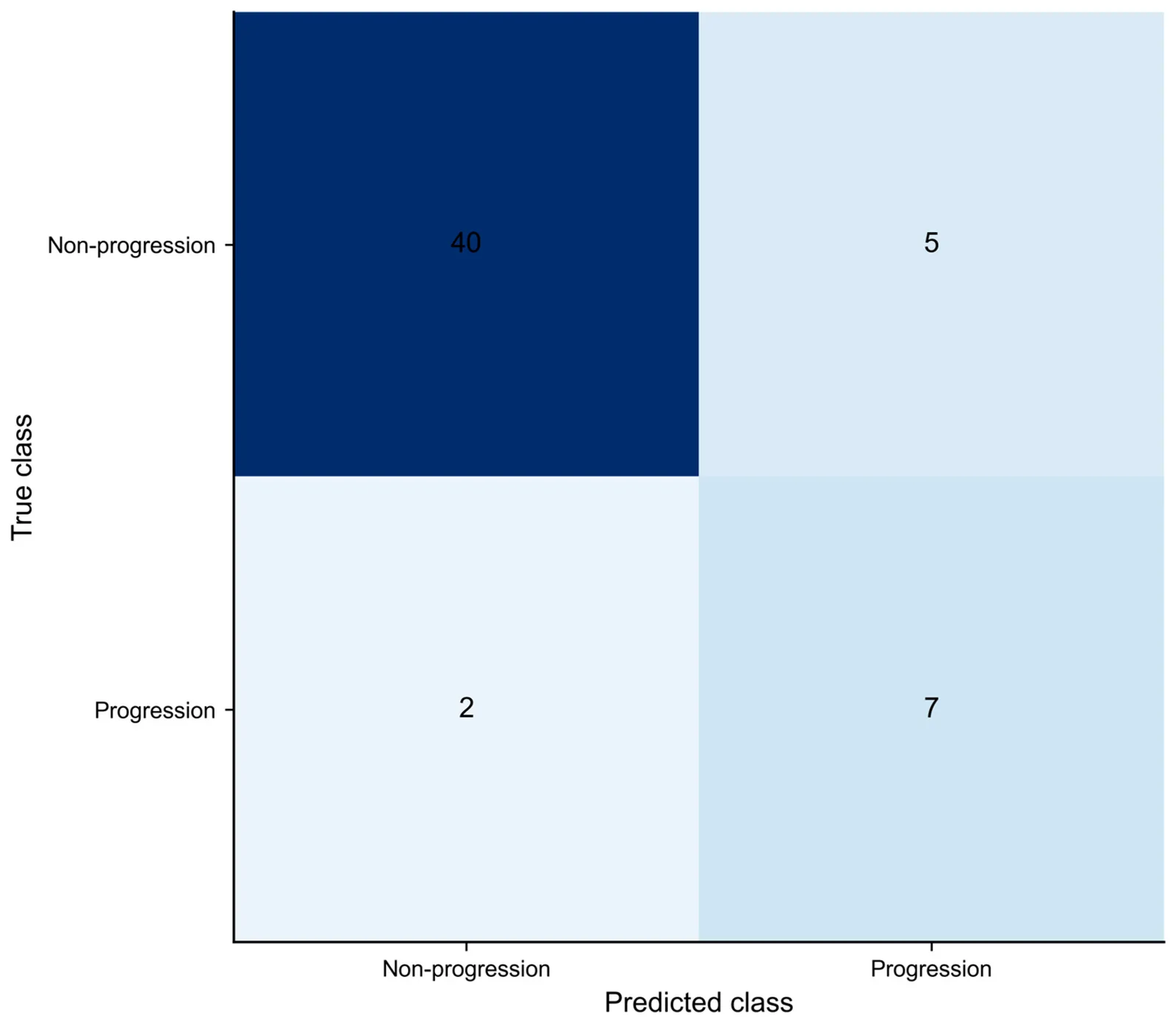
Confusion matrix for the primary ΔT1C-WI progression model based on averaged patient-level out-of-fold predictions at the prespecified probability threshold of 0.50 (true positive, 7; false negative, 2; true negative, 40; false positive, 5).

The mean receiver operating characteristic curve for the primary ΔT1C-WI model is shown in Figure 4, and comparison of AUC values across the binary radiomics models is shown in Figure 5. Using averaged patient-level out-of-fold probabilities, the primary model achieved an AUC of 0.914 (95% CI, 0.822–0.980), sensitivity of 77.8% (95% CI, 40.0–97.2%), specificity of 88.9% (95% CI, 75.9–96.3%), and accuracy of 87.0% (95% CI, 75.1–94.6%) at the prespecified probability threshold of 0.50. The Brier score was 0.112. The calibration curve indicated some overestimation of progression probability (Figure 6). The confusion matrix included 40 true negatives, 5 false positives, 2 false negatives, and 7 true positives (Figure 7). Exploratory decision curve analysis showed greater net benefit than the treat-all and treat-none strategies across threshold probabilities of approximately 0.07–0.66. Given the limited number of progression events, these findings should be interpreted cautiously.

### 3.5. Feature Stability Analysis

Shape descriptors were the most consistently retained predictors, while texture-based heterogeneity features provided complementary information. Least axis length, major axis length, and minor axis length were selected in 98%, 96%, and 94% of the 50 outer cross-validation models, respectively. NGTDM strength, the GLCM maximal correlation coefficient, NGTDM coarseness, and NGTDM busyness were also frequently retained. Detailed feature-stability results are provided in Supplementary Table S6.

An exploratory elastic-net nomogram-style representation showing the relative contribution of the selected ΔT1C-WI features is provided as Supplementary Figure S1. This representation was derived from the full-cohort descriptive refit and was not used for performance estimation.

## 4. Discussion

In this study, delta radiomics derived from 6-month post-treatment MRI showed better performance than baseline radiomics for predicting 3-year volumetric progression after GKRS in sellar–parasellar meningiomas. Among the evaluated feature sets, the strongest performance was obtained with the ΔT1C-WI model, which achieved a mean AUC of 0.861 ± 0.065 in repeated nested cross-validation, with a sensitivity of 0.778 and a specificity of 0.889. In contrast, baseline T1C-WI, baseline ADC, and combined baseline models demonstrated lower discrimination. ΔADC radiomics showed moderate predictive performance; however, adding ΔADC features to ΔT1C-WI did not improve model performance. These findings suggest that longitudinal changes on contrast-enhanced T1-weighted imaging may capture treatment-related information that is more relevant to later volumetric progression than baseline imaging characteristics alone. The present study extends previous meningioma radiomics research by focusing on longitudinal post-treatment changes in a specifically defined sellar–parasellar cohort and by using 3-year volumetric progression as the prediction target.

Models incorporating longitudinal radiomic changes showed higher performance than those based only on baseline imaging characteristics, suggesting that treatment-related changes in imaging features may provide more informative signals for response assessment than pre-treatment tumor characteristics alone. This is clinically relevant because GKRS-treated meningiomas often show slow growth and delayed volumetric response, which limits early response assessment on routine follow-up imaging. Delta radiomics may therefore offer a quantitative approach to characterize interval imaging changes and may help support individualized follow-up strategies after radiosurgery.

DWI has been investigated as an imaging marker after GKRS in brain metastases, vestibular schwannomas, and meningiomas [6,12,13,23], but its value for treatment response assessment in sellar–parasellar meningiomas remains less defined. DWI enables characterization of tumor microstructure without contrast administration and has therefore been proposed as a potential marker for treatment response [24]. Histopathological studies have shown that ADC values may reflect treatment-induced cellular loss, cytoplasmic shrinkage, and necrosis [25] and that higher post-treatment ADC values typically reflect reduced cellularity [26,27]. Conversely, restricted diffusion in untreated or progressing tumors may result from hypoxic microenvironments and reduced extracellular space [28]. In meningiomas, however, ADC values are influenced by extracellular matrix composition, vascularity, hemorrhagic components, and regional heterogeneity, which contribute to variability in diffusion measurements and limit their consistent applicability across studies [29].

GKRS is widely used for recurrent or residual sellar–parasellar meningiomas and provides high tumor control rates, with approximately 90–98% of tumors showing stability or regression after treatment [1,30,31]. Response assessment based on tumor size alone may be misleading, as transient volume increases have been reported within the first year following radiosurgery, sometimes reflecting treatment-induced edema rather than true progression [31,32]. This phenomenon may complicate early follow-up interpretation despite eventual tumor stabilization. At the microstructural level, GKRS induces cytotoxic effects, including necrosis, vascular damage, and reduced cellular density through disruption of membrane integrity and extracellular matrix remodeling [32,33], followed by vascular occlusion and reduced permeability in the subacute phase [30,34]. Such treatment-related alterations expand extracellular spaces and reduce intracellular diffusion restrictions, producing measurable ADC increases that may precede volumetric changes by weeks to months [24]. These mechanisms support the use of longitudinal imaging biomarkers, such as delta radiomic and diffusion-derived features, after radiosurgery.

While conventional radiological follow-up relies on macroscopic volume changes that may take months to become evident, radiomics captures subvisual quantitative features reflecting tumor microstructure and may support earlier assessment of treatment response [35,36]. Radiomics has been shown to predict volumetric response after GKRS in meningiomas, although studies specifically addressing sellar–parasellar meningiomas remain limited. Speckter et al. demonstrated that pre-treatment MRI radiomic features were associated with volumetric response, with AUC values up to 0.81, increasing to 0.88 when combined with Karnofsky Performance Status, and texture features emerged as key predictors [37]. That study evaluated long-term volumetric shrinkage over a mean follow-up of 35.7 months, whereas our study aimed to predict 3-year volumetric progression using delta radiomic features derived from 6-month MRI.

Diffusion-based biomarkers have been explored for assessing radiosurgical response in meningiomas [38,39,40]. Early studies demonstrated associations between pre-treatment diffusion tensor imaging (DTI) parameters and volumetric response after GKRS [38], and subsequent histogram-based DTI analyses further supported the prognostic value of diffusion-derived features. However, these studies primarily evaluated diffusion parameters rather than implementing a comprehensive radiomics pipeline integrated with ML-based predictive modeling [13].

In the present study, we developed a radiomics-based supervised classification model to predict volumetric progression following GKRS using both baseline and longitudinal imaging features. In contrast to prior studies focusing mainly on pre-treatment imaging characteristics, our approach incorporated delta radiomics features reflecting treatment-related changes between pre-treatment and 6-month post-treatment MRI. Delta radiomics quantifies temporal changes in imaging features and may reveal treatment-related tumor alterations that precede or accompany measurable volumetric response [41]. Our findings showed that ΔT1C-WI radiomics provided the highest performance among the evaluated feature sets for identifying tumors at risk of 3-year volumetric progression. Diffusion-derived radiomic features, in contrast, showed moderate predictive ability and did not provide additional benefit beyond contrast-enhanced radiomics. This may be related to the complex biological determinants of ADC in meningiomas and to the 6-month imaging timepoint, at which early diffusion-related changes may have partly stabilized, whereas morphologic and enhancement-related changes captured by T1C-WI may remain more informative. Baseline tumor volume showed limited standalone discrimination and did not meaningfully improve the ΔT1C-WI model, suggesting that the predictive signal was not primarily attributable to initial tumor size.

To our knowledge, this study adds to the limited literature on radiomics-based prediction of radiosurgical outcomes in meningiomas by evaluating 6-month delta radiomics for prediction of 3-year volumetric progression in sellar–parasellar meningiomas.

Morphological heterogeneity in tumors has been recognized as a source of predictive information for radiotherapy resistance [42]. A comprehensive set of radiomic features was extracted from meningioma MRI, including shape, first-order, and higher-order texture descriptors. In the feature stability analysis across the 50 outer-fold models, the most consistently retained ΔT1C-WI predictors were shape descriptors, including least axis length, major axis length, and minor axis length, which may reflect early longitudinal changes in lesion geometry after radiosurgery. Texture-based features, including NGTDM strength, coarseness, and busyness, and GLCM maximal correlation coefficient, were also frequently retained, indicating that changes in intratumoral heterogeneity provided complementary predictive information.

NGTDM busyness reflects rapid gray-level changes between neighboring voxels and captures local image complexity. Higher busyness values have previously been associated with less favorable volumetric reduction in meningiomas after radiosurgery in baseline non-contrast T1-WI radiomics, suggesting that increased intratumoral heterogeneity may indicate a more treatment-resistant tumor microenvironment [37]. NGTDM coarseness reflects local image uniformity, with higher values indicating larger homogeneous regions and lower values indicating finer texture patterns and greater spatial variability [43]. The frequent retention of both busyness and coarseness in our model suggests that local gray-level complexity and regional image uniformity contributed to the texture component of the ΔT1C-WI radiomic signal. GLCM maximal correlation coefficient was also frequently retained, reflecting spatial relationships between voxel intensities that may correspond to treatment-related alterations in tissue architecture [43]. Together, these texture features provided complementary information to the dominant shape-based radiomic signal.

Although DWI has been suggested to capture microstructural alterations following radiosurgery [44,45], we hypothesized that integrating ADC-derived features with T1C-WI radiomics would improve response prediction. In our cohort, however, the combined ΔT1C-WI + ΔADC model did not outperform ΔT1C-WI alone, suggesting limited incremental value of ADC-derived features. A recent literature review by Song et al. identified only 13 radiomics studies combining ADC with conventional MRI sequences in meningioma research to date, most of which focused on tumor characterization rather than treatment response prediction [46]. The role of ADC radiomics in predicting post-radiosurgical outcomes, therefore, remains insufficiently explored.

Both biological and technical factors may explain the moderate performance of ADC-derived models in our study. GKRS induces a sequence of microstructural changes, including early cytotoxic effects and transient ADC elevation in the acute and subacute phases, followed by stabilization and structural remodeling [32,33,34]. By the 6-month timepoint in our study, these early diffusion alterations may have partly stabilized, while morphological and enhancement-related changes captured by T1C-WI may have become more prominent. Although a RESOLVE readout-segmented EPI sequence was used to reduce susceptibility-related artifacts, DWI/ADC has lower spatial resolution and signal-to-noise ratio than contrast-enhanced structural MRI and remains susceptible to residual distortion and partial-volume effects in relatively small sellar–parasellar lesions, which may have affected feature stability. Among the sequences evaluated in this study, 6-month ΔT1C-WI radiomics provided the strongest predictive signal for 3-year volumetric progression. Future studies should investigate whether ADC-derived features offer greater value at earlier post-treatment timepoints, when diffusion-related microstructural changes may be more pronounced. However, this possibility remains speculative because earlier post-treatment MRI was not evaluated. The proposed model should be considered an exploratory adjunct to, rather than a replacement for, routine MRI surveillance. Its potential value for individualized follow-up decisions, workflow, and patient management requires external validation and prospective evaluation.

This study has several limitations. The retrospective, single-center design, small cohort, and limited number of progression events may restrict generalizability and increase the risk of residual overfitting despite training-fold-only preprocessing, elastic-net regularization, inverse class-frequency weighting, and repeated nested cross-validation. Manual segmentation may also introduce reader-dependent variability. Although 47 of 107 ΔT1C-WI features achieved an ICC of at least 0.80 in the interobserver subset, intraobserver reproducibility was not assessed. All MRI examinations were acquired on a single scanner, which improved protocol consistency but may limit applicability to other scanners and acquisition protocols. Although a RESOLVE readout-segmented EPI sequence was used to reduce susceptibility-related distortion, residual distortion, lower spatial resolution, and partial-volume effects may still have reduced the stability of ADC-derived features in the sellar–parasellar region [47,48]. Primary and salvage GKRS represent clinically distinct treatment pathways, but the limited number of progression events after stratification precluded robust subgroup-specific prognostic analysis. Findings are therefore interpreted primarily at the cohort level, and the influence of treatment setting warrants evaluation in larger series. In the salvage subgroup, observed delta-radiomic changes may reflect both treatment-related effects and the underlying biology of residual or recurrent disease, which could not be separated in this cohort. Finally, outcome assessment was based on 3-year volumetric change rather than longer-term clinical endpoints; larger prospective multicenter studies with serial imaging and extended follow-up are required to validate and extend these findings.

## 5. Conclusions

This study suggests that delta radiomics derived from 6-month post-treatment contrast-enhanced T1-weighted MRI may help identify sellar–parasellar meningiomas at risk of volumetric progression at 3 years after Gamma Knife radiosurgery. Among the evaluated models, ΔT1C-WI radiomics showed the strongest performance, whereas the addition of ADC-derived features did not improve discrimination. Longitudinal morphologic and texture changes on contrast-enhanced imaging may therefore provide useful information for early risk stratification after radiosurgery. Given the limited number of progression events and the single-center design, these findings require validation in larger independent cohorts before clinical implementation. Prospective studies are also needed to evaluate their potential effect on surveillance strategies and workflow.

## Figures and Tables

**Table 1:** Demographic, clinical, treatment, and volumetric characteristics of the study cohort.

Variable	Value
**Age, years**	
Mean ± SD	54.4 ± 11.8
Median (IQR)	54.5 (47.3–62.0)
Range	30–78
**Sex, n (%)**	
Female	41 (75.9)
Male	13 (24.1)
**Treatment setting, n (%)**	
Primary GKRS	38 (70.4)
Salvage GKRS after surgery	16 (29.6)
**Histopathological confirmation, n (%)**	
Available	16 (29.6)
Radiologically presumed	38 (70.4)
**WHO grade among histologically confirmed tumors, n (%)**	
Grade I	12 (75.0)
Grade II	4 (25.0)
**Tumor location, n (%)**	
Cavernous sinus	19 (35.2)
Sphenoid wing	15 (27.8)
Petroclival confluence	12 (22.2)
Planum sphenoidale	4 (7.4)
Tuberculum sellae	3 (5.6)
Diaphragma sellae	1 (1.9)
**GKRS prescription dose, Gy**	
Median (IQR)	13.0 (12.0–13.8)
Range	9–20
**Tumor volume**	
Baseline volume, cm^3^, mean ± SD	8.0 ± 9.6
Baseline volume, cm^3^, median (range)	5.2 (0.6–51.2)
3-year volume, cm^3^, mean ± SD	7.3 ± 9.0
3-year volume, cm^3^, median (range)	4.9 (0.6–47.5)
Absolute volume change, cm^3^, mean ± SD	−0.73 ± 3.26
Percentage volume change, %, mean ± SD	−8.2 ± 26.3
**Treatment response at 3 years, n (%)**	
Regression (>20% volume decrease)	20 (37.0)
Stable disease (change between −20% and +20%)	25 (46.3)
Progression (>20% volume increase)	9 (16.7)
Local control	45 (83.3)

**Note:** Data are presented as number (percentage), mean ± standard deviation, or median (interquartile range or range), as appropriate. GKRS, Gamma Knife radiosurgery; IQR, interquartile range; SD, standard deviation. WHO grade percentages were calculated among the 16 histopathologically confirmed tumors.

**Table 2:** Performance of binary radiomics models for prediction of 3-year volumetric progression.

Model	AUC	Accuracy	F1 Score	Sensitivity	Specificity	Brier Score
Baseline T1C-WI	0.686 ± 0.117	0.757 ± 0.033	0.392 ± 0.149	0.556	0.844	0.166
Baseline ADC	0.592 ± 0.102	0.669 ± 0.060	0.294 ± 0.124	0.333	0.711	0.198
Baseline T1C-WI + ADC	0.695 ± 0.109	0.765 ± 0.045	0.415 ± 0.125	0.556	0.867	0.165
ΔT1C-WI	0.861 ± 0.065	0.846 ± 0.037	0.579 ± 0.097	0.778	0.889	0.112
ΔADC	0.756 ± 0.064	0.787 ± 0.046	0.457 ± 0.091	0.556	0.867	0.154
ΔT1C-WI + ΔADC	0.747 ± 0.074	0.802 ± 0.033	0.426 ± 0.086	0.444	0.933	0.123

**Note:** AUC, accuracy, and F1 score are presented as mean ± SD across the 10 repeated nested cross-validation runs. Sensitivity, specificity, and Brier score were calculated from averaged patient-level out-of-fold probabilities at a probability threshold of 0.50. AUC, area under the receiver operating characteristic curve; SD, standard deviation.

## Data Availability

An anonymized radiomic feature dataset with outcome labels is provided as Supplementary Data S1 in CSV format. Additional data supporting the findings of this study are not publicly available because of patient privacy, ethical, and institutional restrictions but may be available from the corresponding author upon reasonable request and subject to institutional approval.

## Supplementary Materials

The following supporting information can be downloaded at: https://www.mdpi.com/article/10.3390/diagnostics16152473/s1, Supplementary Tables S1: Interobserver reproducibility of ΔT1C-WI radiomic features; Table S2: Documented reasons for selecting primary GKRS; Table S3: Patient-level details for previously operated patients; Table S4: Exploratory comparison of available GKRS treatment parameters between progression and non-progression groups; Table S5: Bidimensional RANO benchmark and agreement with volumetric classification; Table S6: Top 15 stable ΔT1C-WI features; Figure S1: Nomogram; Supplementary Checklists S1: CLEAR Checklist and Supplementary Checklist S2. TRIPOD+AI Checklist; and Supplementary_Data_S1_Anonymized_Radiomic_Features.

## Abbreviations

The following abbreviations are used in this manuscript:

ADC	Apparent diffusion coefficient
DWI	Diffusion-weighted imaging
EPI	Echo-planar imaging
GKRS	Gamma Knife radiosurgery
GLCM	Gray-level co-occurrence matrix
GLDM	Gray-level dependence matrix
GLRLM	Gray-level run length matrix
GLSZM	Gray-level size zone matrix
ICC	Intraclass correlation coefficient
NGTDM	Neighboring gray tone difference matrix
ROC	Receiver operating characteristic
T1C-WI	Contrast-enhanced T1-weighted imaging
