# Supplementary material for: Early Prediction of Volumetric Progression in Sellar–Parasellar Meningiomas Using Delta Radiomics on 6-Month MRI Following Gamma Knife Radiosurgery
Source: Diagnostics (Basel). 2026 Aug 5;16(15):2473. doi: 10.3390/diagnostics16152473 (PMC13465353; doi:10.3390/diagnostics16152473)
Supplement: Supplementary file 1 [file diagnostics-16-02473-s001.zip › Supplementary_Checklist_S1_CLEAR.pdf]

Supplementary Checklist S1. CLEAR Checklist

Early Prediction of Volumetric Progression in Sellar-Parasellar Meningiomas Using Delta Radiomics on 6-Month MRI Following Gamma Knife Radiosurgery

Checklist basis: Kocak et al., CLEAR, Insights into Imaging 2023;14:75. Status reflects the current clean revised manuscript. “No” indicates that an item is not fully reported or not provided; it is not a quality score.

No.	Section	CLEAR item	Yes	No	N/A	Location / comment
1	Title	Relevant title specifying radiomic methodology	✓			Title, p. 1. Title includes delta radiomics, MRI, and GKRS.
2	Abstract	Structured summary with relevant information	✓			Abstract, p. 2. Structured abstract reports cohort, methods, validation, performance, and conclusion.
3	Keywords	Relevant radiomics keywords	✓			Keywords, p. 2. Includes radiomics, delta radiomics, MRI-related and machine-learning terms.
4	Introduction	Scientific or clinical background	✓			Introduction, pp. 2–4. Clinical context, prior evidence, and knowledge gap are described.
5	Introduction	Rationale for using a radiomic approach	✓			Introduction, pp. 2–4. Rationale for longitudinal radiomics and limitations of size-based assessment are stated.
6	Introduction	Study objective(s)	✓			Introduction, p. 4. Primary and secondary aims are explicitly stated.
7	Study design	Adherence to guidelines or checklists	✓			Methods 2.11, p. 14. CLEAR, TRIPOD+AI, and RQS are stated.
8	Study design	Ethical details, consent, and data protection	✓			Methods 2.1, p. 4; Statements, p. 26. IRB approval, waiver, anonymization, and restrictions are reported.
9	Study design	Sample-size determination	✓			Methods 2.1, p. 5. All consecutive eligible patients were included; no a priori calculation.
10	Study design	Study nature	✓			Methods 2.1, p. 4. Retrospective single-center cohort.
11	Study design	Eligibility criteria and selection process	✓			Methods 2.1, pp. 4–5. Inclusion, exclusion, and consecutive selection are reported.
12	Study design	Flowchart for technical pipeline	✓			Figure 2, pp. 6–7. Radiomics pipeline and validation workflow are shown.
13	Data	Data source	✓			Methods 2.1, p. 4. Institutional radiosurgery database/private institutional data.
14	Data	Data overlap with previous publications		✓		Not reported. No statement on whether any dataset overlap exists.
15	Data	Data split methodology and leakage prevention	✓			Methods 2.9–2.10, pp. 12–13. Patient-level repeated nested stratified CV and training-fold-only preprocessing are described.
16	Data	Imaging protocol and acquisition parameters	✓			Methods 2.3, pp. 7–8. Scanner, sequences, timing, parameters, voxel dimensions, and ADC calculation are reported.
17	Data	Definition of non-radiomic predictor variables	✓			Methods 2.10–2.11, pp. 13–14. Baseline tumor volume and treatment parameters are defined as secondary/non-radiomic analyses.
18	Data	Definition and rationale of reference standard/outcome	✓			Methods 2.6, pp. 8–10. Three-year volumetric endpoint and secondary RANO benchmark are defined.
19	Segmentation	Segmentation strategy	✓			Methods 2.4, p. 8; Figure 3. Manual 3D whole-tumor segmentation, software/version,

No.	Section	CLEAR item	Yes	No	N/A	Location / comment
						no registration, and example are reported.
20	Segmentation	Operators and consensus procedure	✓			Methods 2.4, p. 8. Two neuroradiologists with >10 years experience; consensus final segmentations.
21	Pre-processing	Image pre-processing details	✓			Methods 2.7, p. 11. Normalization, resampling, discretization, bias correction, pre-cropping, and settings are described.
22	Pre-processing	Resampling method and parameters	✓			Methods 2.7, p. 11. T1C-WI B-spline to 1 mm isotropic; ADC linear to 2 mm isotropic.
23	Pre-processing	Discretization method and parameters	✓			Methods 2.7, p. 11. Fixed bin width 5 for T1C-WI and 25 for ADC.
24	Pre-processing	Image types used for feature extraction	✓			Methods 2.7, p. 11. Original image type only; wavelet and LoG excluded.
25	Feature extraction	Feature extraction method	✓			Methods 2.7, p. 11. SlicerRadiomics/PyRadiomics, IBSI framework, 3D extraction, software versions.
26	Feature extraction	Feature classes	✓			Methods 2.7, p. 11. Shape, first-order, GLCM, GLRLM, GLSZM, GLDM, and NGTDM with counts.
27	Feature extraction	Number of features	✓			Methods 2.7, p. 11. 107 features per sequence/timepoint before volume-feature exclusion.
28	Feature extraction	Statement that remaining parameters used defaults		✓		Not reported. Exact modified settings are reported, but no explicit default-configuration statement.
29	Data preparation	Handling and amount of missing data		✓		Methods 2.9, p. 12. Median imputation is described, but the amount of missing data is not reported.
30	Data preparation	Class imbalance and handling	✓			Methods 2.9, p. 12; Results 3.1, p. 15. 9:45 imbalance, class weighting, stratification, and no resampling are reported.
31	Data preparation	Segmentation reliability analysis		✓		Methods 2.5, p. 8; Results 3.2, p. 15. Interobserver ICC is reported; intraobserver analysis and training-set-only restriction were not performed/stated.
32	Data preparation	Feature scaling details	✓			Methods 2.9–2.10, pp. 12–13. Z-score standardization within training folds.
33	Data preparation	Dimension reduction details	✓			Methods 2.9–2.10, pp. 12–13. Variance filter, mutual information up to 80, correlation threshold >0.90, all within training folds.
34	Modeling	Algorithm and software details	✓			Methods 2.9 and 2.11, pp. 12–14. Elastic-net logistic regression and package versions are reported.
35	Modeling	Training and hyperparameter tuning details	✓			Methods 2.10, pp. 12–13. Grid, solver, nested loops, repetitions, and refitting are described.
36	Modeling	Detection and handling of confounders		✓		Not reported. No formal confounder-identification or covariate-adjustment strategy.
37	Modeling	Model selection strategy	✓			Methods 2.10, pp. 12–13. Inner-loop grid search and held-out outer-fold evaluation; primary model predefined.
38	Evaluation	Internal/external testing technique	✓			Methods 2.10, pp. 12–13. Repeated nested internal validation; external validation not performed and is acknowledged.

No.	Section	CLEAR item	Yes	No	N/A	Location / comment
39	Evaluation	Performance metrics and rationale	✓			Methods 2.11, pp. 13–14. AUC primary; accuracy, F1, sensitivity, specificity, Brier score also reported.
40	Evaluation	Uncertainty evaluation and measures	✓			Methods 2.11, p. 13; Results 3.3–3.4, pp. 16–18. SDs, bootstrap/binomial CIs, calibration, and Brier score.
41	Evaluation	Statistical performance comparison	✓			Methods 2.11, p. 13; Results 3.3, p. 18. Paired DeLong comparisons and p-values are reported.
42	Evaluation	Comparison with non-radiomic/combined methods and utility	✓			Methods 2.10–2.11, pp. 13–14; Results 3.3–3.4, pp. 16–18. Baseline volume benchmark, combined models, RANO benchmark, and DCA are reported.
43	Evaluation	Interpretability/explainability methods	✓			Methods 2.11, pp. 13–14; Results 3.5, p. 20; Figure S1. Feature stability and descriptive coefficient-based nomogram-style representation.
44	Results	Baseline demographic and clinical characteristics	✓			Table 1 and Results 3.1, pp. 9–15. Cohort characteristics and outcome groups are reported; fixed data partitions are not applicable.
45	Results	Eligibility flowchart	✓			Figure 1, pp. 5–6. Screening, exclusions, final cohort, treatment groups, and outcomes.
46	Results	Feature statistics and reproducibility/selection	✓			Results 3.2 and 3.5, pp. 15 and 20; Tables S1 and S6. ICC and feature selection-frequency results are provided.
47	Results	Model performance evaluation		✓		Results 3.3–3.4, pp. 16–19. Extensive internal-validation metrics and calibration are reported, but no-information rate is not explicitly provided.
48	Results	Comparison with non-radiomic and combined approaches	✓			Results 3.3–3.4, pp. 16–19. Comparative AUCs, uncertainty, DeLong p-values, confusion matrix, baseline-volume benchmark, and DCA are reported.
49	Discussion	Overview of important findings	✓			Discussion, pp. 20–21. Main findings and exploratory context are summarized.
50	Discussion	Comparison with previous work	✓			Discussion, pp. 21–24. Relevant prior radiosurgery/radiomics studies and differences are discussed.
51	Discussion	Practical implications and translation	✓			Discussion, pp. 24–25; Conclusions, p. 25. Adjunctive role, external validation, prospective evaluation, and workflow implications.
52	Discussion	Strengths and limitations	✓			Discussion, pp. 24–25. Bias, overfitting, segmentation, scanner, ADC, subgroup, and generalizability limitations.
53	Open science	Sharing images and segmentation data [recommended]		✓		Data Availability, p. 26. Not shared due to privacy, ethical, and institutional restrictions.
54	Open science	Sharing radiomic feature data	✓			Data Availability, p. 26; Supplementary Data S1. Anonymized extracted radiomic features and outcome labels are provided.
55	Open science	Sharing pre-processing scripts or settings	✓			Methods 2.7, p. 11. Complete textual description of preprocessing and feature-extraction settings is provided; no separate YAML file.
56	Open science	Sharing modeling source code		✓		Not deposited in a public repository or included in the supplementary package. Available from the corresponding author upon reasonable request.
57	Open science	Sharing final model files		✓		Not provided. No deployable final model file is provided; performance is based on internal validation.
58	Open science	Sharing a ready-to-use system [recommended]			✓	Not applicable. No clinical/deployment tool was developed for this exploratory study.
