# Supplementary material for: Early Prediction of Volumetric Progression in Sellar–Parasellar Meningiomas Using Delta Radiomics on 6-Month MRI Following Gamma Knife Radiosurgery
Source: Diagnostics (Basel). 2026 Aug 5;16(15):2473. doi: 10.3390/diagnostics16152473 (PMC13465353; doi:10.3390/diagnostics16152473)
Supplement: Supplementary file 1 [file diagnostics-16-02473-s001.zip › Supplementary_Checklist_S2_TRIPOD_AI.pdf]

Supplementary Checklist S2. TRIPOD+AI Checklist

Early Prediction of Volumetric Progression in Sellar-Parasellar Meningiomas Using Delta Radiomics on 6-Month MRI Following Gamma Knife Radiosurgery

Checklist basis: Collins et al., TRIPOD+AI, BMJ 2024;385:e078378. This is a development study using internal repeated nested cross-validation; items applicable only to external evaluation (E) are marked N/A. Status reflects the current clean revised manuscript. “No” indicates that an item is not fully reported or not provided; it is not a quality score.

No.	Section	TRIPOD+AI item	Yes	No	N/A	Location / comment
1	Title	Identifies the study as developing a prediction model, the target population, and the outcome to be predicted	✓			Title page. Title specifies delta radiomics, sellar-parasellar meningiomas, and volumetric progression as the predicted outcome.
2	Abstract	Structured abstract summarizing objectives, methods, results, and conclusions	✓			Abstract. Structured abstract reports cohort, methods, validation approach, performance, and conclusion.
3a	Introduction	Healthcare context and rationale for developing the model, with references to existing models	✓			Introduction, Section 1. Clinical background, prior radiomics evidence, and knowledge gap are described.
3b	Introduction	Target population and intended purpose of the model in the care pathway, including intended users		✓		Target population described (Section 1, Section 2.1) but intended clinical users and placement in the care pathway are not explicitly stated.
3c	Introduction	Known health inequalities between sociodemographic groups		✓		Not addressed.
4	Introduction	Study objectives, specifying model development and/or evaluation	✓			Introduction, Section 1, final paragraph. States objective to develop and internally validate the model.
5a	Methods – Data	Source of data for development (and evaluation, if applicable), rationale, and representativeness	✓			Methods, Section 2.1. Retrospective single-center institutional radiosurgery database. No separate external evaluation dataset; performance was estimated only through internal repeated nested cross-validation.
5b	Methods – Data	Dates of participant accrual and end of follow-up		✓		Not reported. Accrual start/end dates are not stated in Section 2.1.
6a	Methods – Participants	Study setting, number and location of centres	✓			Methods, Section 2.1/2.3. Single-center study (Gazi University), single 1.5T MRI scanner.
6b	Methods – Participants	Eligibility criteria for participants	✓			Methods, Section 2.1. Inclusion and exclusion criteria are listed.
6c	Methods – Participants	Treatments received and how handled in the analysis	✓			Methods, Section 2.2 (Radiosurgery Treatment Protocol); treatment setting (primary vs. salvage GKRS) addressed in Section 2.1.
7	Methods – Data preparation	Data pre-processing and quality checking	✓			Methods, Section 2.7 (image preprocessing) and Section 2.9 (pipeline preprocessing steps).
8a	Methods – Outcome	Outcome definition, time horizon, method of assessment, and rationale	✓			Methods, Section 2.6. Volumetric progression at 3 years defined with threshold and literature rationale [4,16,17].
8b	Methods – Outcome	Qualifications of outcome assessors where subjective interpretation is required			✓	Outcome derived from volumetric segmentation; assessor qualifications reported under segmentation (Section 2.4–2.5) rather than as a separate

						outcome-assessment step.
8c	Methods – Outcome	Blinding of outcome assessment		✓		Not explicitly stated whether outcome measurement was performed blind to other data.
9a	Methods – Predictors	Choice of initial predictors and any pre-selection before model building	✓			Methods, Section 2.7 (107 baseline radiomic features) and Section 2.9 (feature selection steps).
9b	Methods – Predictors	Definition of all predictors, including how and when measured	✓			Methods, Sections 2.7–2.8 (feature extraction and delta radiomics calculation).
9c	Methods – Predictors	Qualifications of assessors for predictors requiring subjective interpretation	✓			Methods, Section 2.4–2.5. Manual segmentation performed by two neuroradiologists; consensus and reproducibility subset described.
10	Methods – Sample size	Explanation of how study size was determined and justification of sufficiency	✓			Methods, Section 2.1. States that all consecutive eligible patients were included and that no formal a priori sample-size calculation was performed.
11	Methods – Missing data	How missing data were handled	✓			Methods, Section 2.9. Median imputation applied within the training-fold-only pipeline.
12a	Methods – Analysis	How data were used/partitioned across the analysis	✓			Methods, Sections 2.9–2.10. Repeated nested cross-validation with stratified inner/outer folds.
12b	Methods – Analysis	Handling of predictors (rescaling, transformation, standardization)	✓			Methods, Section 2.9. Z-score standardization within the training-fold pipeline.
12c	Methods – Analysis	Model type and rationale, model-building steps, hyperparameter tuning, internal validation method	✓			Methods, Sections 2.9–2.10. Elastic-net logistic regression; grid search over C and l1_ratio; nested cross-validation.
12d	Methods – Analysis	Heterogeneity in model parameters/performance across clusters (e.g., centres)			✓	Single-center study; no multi-center clustering to evaluate.
12e	Methods – Analysis	Performance measures and plots used, and rationale, including model comparisons	✓			Methods, Section 2.11. AUC, accuracy, F1, sensitivity/specificity, Brier score, calibration, DeLong tests, decision curve analysis.
12f	Methods – Analysis	Model updating (e.g., recalibration) arising from evaluation			✓	Applies to external evaluation studies; this is a development study with internal cross-validation only.
12g	Methods – Analysis	How model predictions were calculated for evaluation (formula, code, API)			✓	Applies to external evaluation studies; not applicable to this internal development study.
13	Methods – Class imbalance	Class imbalance methods used, rationale, and any recalibration	✓			Methods, Section 2.9. Inverse class-frequency weighting; no synthetic over/undersampling.
14	Methods – Fairness	Approaches used to address model fairness and rationale		✓		Not addressed; no subgroup fairness analysis (e.g., by sex, age) reported.
15	Methods – Model output	Model output type and rationale/thresholds for any classification	✓			Methods, Section 2.11. Binary classification at a prespecified probability threshold of 0.50.
16	Methods – Development vs. evaluation	Differences between development and evaluation data			✓	No separate external evaluation dataset; internal cross-validation only.
17	Methods – Ethical approval	Ethics committee approval and informed consent/waiver	✓			Institutional Review Board Statement. Gazi University IRB approval (protocol 2026-425); consent waived for retrospective design.
18a	Open science – Funding	Source of funding and role of funders	✓			Funding statement. No external funding; APC funded by authors.
18b	Open science –	Conflicts of interest and financial	✓			Conflicts of Interest statement.

	Conflicts of interest	disclosures				Authors declare no conflicts of interest.
18c	Open science – Protocol	Availability of study protocol		✓		No study protocol was registered prior to data analysis, consistent with the retrospective design of this study.
18d	Open science – Registration	Study registration details, or statement that not registered		✓		No study registration was performed, consistent with the retrospective design of this study.
18e	Open science – Data sharing	Availability of study data	✓			Data Availability Statement. Anonymized radiomic feature dataset with outcome labels provided as Supplementary Data S1; additional data available from corresponding author upon reasonable request.
18f	Open science – Code sharing	Availability of analytical code		✓		Not reported in the manuscript; analytical code was not deposited in a public repository. Available from the corresponding author upon reasonable request.
19	Patient & public involvement	Details of patient/public involvement, or statement of none		✓		Not reported.
20a	Results – Participants	Flow of participants through the study; diagram if helpful	✓			Figure 1 (patient-selection flowchart) and Figure 2 (study workflow).
20b	Results – Participants	Characteristics of the cohort, including predictors, outcomes, and missing data	✓			Table 1; Results, Section 3.1.
20c	Results – Participants	Comparison of predictor/outcome distributions between development and evaluation data			✓	No separate external evaluation dataset.
21	Results – Model development	Number of participants and outcome events in each analysis	✓			Results, Section 3.1. 54 patients; 9 progression / 45 non-progression for the primary binary analysis.
22	Results – Model specification	Full prediction model details enabling third-party evaluation/implementation		✓		An exploratory nomogram-style relative-weighting figure of the full-cohort refit is provided (Section 3.5), but full model coefficients, code, or an API for independent implementation are not shared.
23a	Results – Model performance	Performance estimates with confidence intervals; plots to aid presentation	✓			Results, Section 3.3–3.4; Table 2; Figures 4–7. AUC with 95% CI, sensitivity, specificity, accuracy, Brier score, calibration curve.
23b	Results – Model performance	Heterogeneity in performance across clusters			✓	Single-center study.
24	Results – Model updating	Results of any model updating			✓	No external validation/updating performed.
25	Discussion – Interpretation	Overall interpretation of results in context of objectives and prior studies	✓			Discussion, Section 4.
26	Discussion – Limitations	Study limitations and their effect on bias, uncertainty, and generalizability	✓			Discussion, Section 4, limitations paragraph. Retrospective single-center design, small cohort, limited progression events, segmentation variability, single-scanner acquisition, and other limitations discussed.
27a	Discussion – Usability	Handling of poor-quality or unavailable input data at implementation		✓		Not discussed.
27b	Discussion – Usability	User interaction and expertise required to use the model		✓		Not discussed.
27c	Discussion – Usability	Next steps for future research, applicability, and generalizability	✓			Discussion/Conclusions, Section 4–5. Need for external validation

						and prospective multicenter studies stated.
