# Supplementary material for: Early Prediction of Volumetric Progression in Sellar–Parasellar Meningiomas Using Delta Radiomics on 6-Month MRI Following Gamma Knife Radiosurgery
Source: Diagnostics (Basel). 2026 Aug 5;16(15):2473. doi: 10.3390/diagnostics16152473 (PMC13465353; doi:10.3390/diagnostics16152473)
Supplement: Supplementary file 1 [file diagnostics-16-02473-s001.zip › Supplementary_Materials.pdf]

Supplementary Materials

Manuscript ID: diagnostics-4413981

Early Prediction of Volumetric Progression in Sellar-Parasellar Meningiomas Using Delta Radiomics on 6-Month MRI Following Gamma Knife Radiosurgery

Supplementary Table S1. Interobserver reproducibility of ΔT1C-WI radiomic features

ICC(2,1): two-way random-effects, absolute-agreement, single-measurement model; n = 15. Features are sorted in descending ICC order. MeshVolume and VoxelVolume were excluded from modeling because they directly represent tumor volume. Forty-seven of the 107 evaluated features met the ICC ≥0.80 threshold after exclusion of these direct volume features. † Direct volume feature excluded from modeling.

Feature	n	ICC(2,1)	Pearson r	Mean absolute difference
original|glcm|Contrast	15	0.995	0.995	19.47
original|glcm|SumSquares	15	0.992	0.992	18.74
original|glcm|ClusterTendency	15	0.989	0.990	55.72
original|glcm|DifferenceVariance	15	0.986	0.986	11.45
original|ngtdm|Contrast	15	0.985	0.985	0.047
original|firstorder|InterquartileRange	15	0.984	0.984	41.68
original|glcm|ClusterProminence	15	0.983	0.983	3.889e+05
original|firstorder|RobustMeanAbsoluteDeviation	15	0.976	0.977	18.88
original|glcm|Idmn	15	0.975	0.974	0.003
original|glcm|DifferenceAverage	15	0.975	0.975	0.930
original|gldm|SmallDependenceHighGrayLevelEmphasis	15	0.975	0.975	79.06
original|glcm|Autocorrelation	15	0.972	0.971	134.9
original|gldm|GrayLevelVariance	15	0.969	0.970	29.66
original|firstorder|Variance	15	0.969	0.970	18547
original|glrlm|ShortRunHighGrayLevelEmphasis	15	0.968	0.966	152.8
original|gldm|HighGrayLevelEmphasis	15	0.967	0.965	159.1
original|glrlm|HighGrayLevelRunEmphasis	15	0.966	0.964	161.9
original|glrlm|GrayLevelVariance	15	0.965	0.967	31.05
original|gldm|DependenceNonUniformityNormalized	15	0.956	0.969	0.018
original|glcm|Idn	15	0.954	0.952	0.008
original|ngtdm|Complexity	15	0.951	0.952	2871
original|glszm|HighGrayLevelZoneEmphasis	15	0.950	0.949	190.0
original|glszm|SmallAreaHighGrayLevelEmphasis	15	0.946	0.946	158.9
original|glszm|SmallAreaLowGrayLevelEmphasis	15	0.941	0.945	0.004
original|firstorder|MeanAbsoluteDeviation	15	0.940	0.944	31.31
original|glrlm|LongRunHighGrayLevelEmphasis	15	0.935	0.931	231.6
original|glcm|ClusterShade	15	0.926	0.929	3036
original|glszm|GrayLevelVariance	15	0.921	0.926	45.52
original|firstorder|90Percentile	15	0.915	0.916	158.9
original|gldm|GrayLevelNonUniformity	15	0.911	0.927	197.2
original|firstorder|Energy	15	0.908	0.923	1.037e+09
original|glcm|SumAverage	15	0.907	0.904	7.227
original|glcm|JointAverage	15	0.907	0.904	3.613
original|firstorder|TotalEnergy	15	0.899	0.892	1.325e+09
original|glszm|ZonePercentage	15	0.888	0.908	0.057
original|gldm|SmallDependenceEmphasis	15	0.888	0.905	0.048
original|firstorder|Median	15	0.884	0.877	102.3
original|glszm|SizeZoneNonUniformity	15	0.880	0.878	316.2
original|firstorder|RootMeanSquared	15	0.879	0.877	125.9
original|glcm|Imc1	15	0.873	0.877	0.036

Feature	n	ICC(2,1)	Pearson r	Mean absolute difference
original|firstorder|Mean	15	0.869	0.865	119.1
original|gldm|DependenceNonUniformity	15	0.861	0.861	348.6
original|glrlm|GrayLevelNonUniformity	15	0.843	0.877	155.0
original|firstorder|Entropy	15	0.820	0.816	0.327
original|glcm|SumEntropy	15	0.817	0.809	0.313
original|gldm|LargeDependenceLowGrayLevelEmphasis	15	0.816	0.853	0.399
original|glcm|DifferenceEntropy	15	0.806	0.807	0.346
original|glcm|InverseVariance	15	0.798	0.810	0.044
original|glszm|LowGrayLevelZoneEmphasis	15	0.791	0.865	0.010
original|glcm|Id	15	0.766	0.755	0.049
original|glrlm|RunEntropy	15	0.762	0.755	0.277
original|shape|Maximum2DDiameterColumn	15	0.758	0.761	6.201
original|ngtdm|Busyness	15	0.754	0.761	0.720
original|glszm|LargeAreaLowGrayLevelEmphasis	15	0.744	0.906	46.18
original|glrlm|GrayLevelNonUniformityNormalized	15	0.735	0.747	0.020
original|glcm|Idm	15	0.725	0.712	0.058
original|firstorder|Uniformity	15	0.722	0.774	0.029
original|glrlm|RunLengthNonUniformity	15	0.717	0.729	1601
original|ngtdm|Strength	15	0.702	0.749	3.933
original|glcm|JointEntropy	15	0.700	0.686	0.643
original|glszm|GrayLevelNonUniformity	15	0.697	0.705	30.03
original|firstorder|Maximum	15	0.695	0.753	333.5
original|glszm|ZoneEntropy	15	0.681	0.672	0.209
original|glcm|Imc2	15	0.670	0.695	0.074
original|firstorder|Range	15	0.666	0.726	322.1
original|firstorder|10Percentile	15	0.655	0.640	105.4
original|shape|Maximum2DDiameterRow	15	0.647	0.659	9.200
original|glrlm|RunLengthNonUniformityNormalized	15	0.645	0.633	0.053
original|gldm|DependenceEntropy	15	0.644	0.687	0.238
original|firstorder|Minimum	15	0.644	0.679	67.67
original|glcm|JointEnergy	15	0.613	0.752	0.020
original|glszm|ZoneVariance	15	0.599	0.763	4173
original|glszm|LargeAreaEmphasis	15	0.598	0.762	4208
original|glcm|MaximumProbability	15	0.594	0.742	0.040
original|shape|SurfaceArea	15	0.581	0.565	757.6
original|glrlm|LongRunLowGrayLevelEmphasis	15	0.580	0.678	0.024
original|gldm|DependenceVariance	15	0.579	0.687	4.348
original|glrlm|RunPercentage	15	0.579	0.593	0.047
original|shape|Flatness	15	0.577	0.639	0.081
original|glrlm|ShortRunEmphasis	15	0.564	0.562	0.032
original|glszm|SizeZoneNonUniformityNormalized	15	0.559	0.548	0.055
original|shape|Maximum2DDiameterSlice	15	0.551	0.594	13.27
original|gldm|LargeDependenceEmphasis	15	0.540	0.687	19.19
original|glcm|MCC	15	0.538	0.557	0.098
original|glszm|LargeAreaHighGrayLevelEmphasis	15	0.512	0.665	4.871e+05
original|shape|Maximum3DDiameter	15	0.493	0.507	12.42
original|firstorder|Kurtosis	15	0.484	0.636	3.265
original|shape|Sphericity	15	0.440	0.456	0.074
original|glszm|SmallAreaEmphasis	15	0.435	0.436	0.047
original|glrlm|LowGrayLevelRunEmphasis	15	0.417	0.508	0.010
original|firstorder|Skewness	15	0.413	0.434	0.678
original|gldm|LowGrayLevelEmphasis	15	0.413	0.490	0.010
original|shape|MinorAxisLength	15	0.402	0.400	2.448

Feature	n	ICC(2,1)	Pearson r	Mean absolute difference
original|glrlm|ShortRunLowGrayLevelEmphasis	15	0.394	0.478	0.008
original|glcm|Correlation	15	0.347	0.350	0.086
original|glszm|GrayLevelNonUniformityNormalized	15	0.327	0.332	0.017
original|shape|VoxelVolume†	15	0.325	0.338	1622
original|shape|LeastAxisLength	15	0.322	0.379	1.693
original|shape|MeshVolume†	15	0.308	0.322	1648
original|glrlm|LongRunEmphasis	15	0.277	0.441	0.555
original|glrlm|RunVariance	15	0.217	0.417	0.285
original|gldm|SmallDependenceLowGrayLevelEmphasis	15	0.204	0.251	0.002
original|gldm|LargeDependenceHighGrayLevelEmphasis	15	0.172	0.290	3839
original|shape|MajorAxisLength	15	0.111	0.121	4.945
original|shape|Elongation	15	0.083	0.086	0.092
original|shape|SurfaceVolumeRatio	15	0.017	0.019	0.154
original|ngtdm|Coarseness	15	-0.259	-0.517	0.004

Supplementary Table S2. Documented reasons for selecting primary GKRS

Documented indication	n	%
High surgical risk related to critical tumor location or anticipated operative morbidity	23	60.5
Comorbidity or advanced age	8	21.1
Patient refusal of surgery	6	15.8
Small tumor volume together with unfavorable location	1	2.6
Total primary GKRS cohort	38	100.0

Supplementary Table S3. Patient-level details for previously operated patients

PatientID	Primary_or_Salvage_GKRS	NumberOfPriorSurgeries	Surgery Type	ExtentOfResection	SimpsonGrade	Residual_or_Recurrent	TimeFromSurgeryToGKRS_months	BaselineTumorVolume_cm3	PrescriptionDose_Gy	TargetVolume_cm3	BinaryOutcome_36m	ResponseGroup_36m
P001	Salvage	1	Left pterional craniotomy and cavernous sinus tumor resectio	STR	IV	Residual	5.68	20.17	14	11.52	0	Stable
P004	Salvage	2	Left pterional craniotomy, tumor resection and optic decompression	STR	IV	Residual	1.31	13.20	13	11.19	0	Regression
P007	Salvage	1	Left pterional/temporal craniotomy and skull-	STR	IV	Residual	5.00	10.38	13	9.78	0	Stable

PatientID	Primary_or_Salvage_GKRS	NumberOfPriorSurgeries	SurgeryType	ExtentOfResection	SimpsonGrade	Residual_or_Recurrent	TimeFromSurgeryToGKRS_months	BaselineTumorVolume_cm3	PrescriptionDose_Gy	TargetVolume_cm3	BinaryOutcome_36m	ResponseGroup_36m
			base tumor resection									
P008	Salvage	1	Left pterional craniotomy, tumor resection and optic decompression	STR	IV	Residual	5.42	45.42	15	10.81	0	Regression
P012	Salvage	2	Left pterional craniotomy, tumor resection and optic decompression	STR	IV	Recurrence	43.00	5.14	12	3.79	1	Progression
P013	Salvage	1	Right pterional frontotemporal craniotomy and sellar–parasellar tumor resection	STR	IV	Residual	1.00	4.50	16	4.42	1	Progression
P015	Salvage	1	Right pterional craniotomy and sellar–parasellar tumor resection	STR	IV	Residual	12.00	6.89	9	4.92	0	Stable
P016	Salvage	1	Left pterional craniotomy, tumor resection and optic decompression	STR	IV	Residual	3.00	5.26	14	4.87	0	Regression
P018	Salvage	2	transsphenoidal resection of tuberculum sellae	STR	IV	Residual	2.00	0.59	20	0.71	1	Progression

PatientID	Primary_or_Salvage_GKRS	NumberOfPriorSurgeries	SurgeryType	ExtentOfResection	SimpsonGrade	Residual_or_Recurrent	TimeFromSurgeryToGKRS_months	BaselineTumorVolume_cm3	PrescriptionDose_Gy	TargetVolume_cm3	BinaryOutcome_36m	ResponseGroup_36m
			meningiomas									
P019	Salvage	1	transsphenoidal resection of tuberculum sellae meningiomas	STR	IV	Residual	15.00	11.29	11	8.04	0	Stable
P025	Salvage	1	Right pterional craniotomy and sphenoid-wing meningioma resection	GTR	II	Recurrence	11.00	1.54	13	1.45	0	Regression
P028	Salvage	2	Left pterional craniotomy and cavernous sinus tumor resectio	STR	IV	Residual	5.50	18.42	14	12.12	1	Progression
P030	Salvage	1	Right pterional craniotomy and sphenoid-wing meningioma resection	STR	IV	Recurrence	42.00	8.85	13	5.98	0	Regression
P046	Salvage	1	left pterional craniotomy and sphenoid-wing meningioma resection	STR	II	Residual	2.00	7.66	14	6.58	0	Regression
P050	Salvage	1	Right pterional craniotomy, subtotal resection and optic canal	STR	IV	Residual	4.00	5.59	13	5.04	0	Regression

PatientID	Primary_or_Salvage_GKRS	NumberOfPriorSurgeries	Surgery Type	ExtentOfResection	SimpsonGrade	Residual_or_Recurrent	TimeFromSurgeryToGKRS_months	BaselineTumorVolume_cm3	PrescriptionDose_Gy	TargetVolume_cm3	BinaryOutcome_36m	ResponseGroup_36m
			decompression									
P052	Salvage	1	right pterional/temporal craniotomy and skull-base tumor resection	STR	IV	Residual	2.00	9.09	11	8.28	0	Stable

Supplementary Table S4. Exploratory comparison of available GKRS treatment parameters between progression and non-progression groups

Treatment parameter	Progression (n = 9), median (IQR)	Non-progression (n = 45), median (IQR)	p value
Prescription dose, Gy	15.0 (12.0–16.0)	13.0 (12.0–13.0)	0.020
Maximum dose, Gy	29.1 (26.2–30.2)	24.4 (22.4–27.1)	0.004
Prescription isodose line, %	50 (50–50)	50 (50–50)	1.000
Target volume, cm³	4.36 (2.82–4.42)	4.73 (2.03–8.04)	0.313
Number of shots	11 (10–13)	15 (12–23)	0.030

Values are presented as median (interquartile range). Groups were compared using exploratory Mann–Whitney U tests. These treatment parameters were not included in the radiomics models. IQR, interquartile range; GKRS, Gamma Knife radiosurgery.

Supplementary Table S5. Bidimensional RANO benchmark and agreement with volumetric classification

All measurements were performed on baseline and 36-month contrast-enhanced T1-weighted MRI. The table below summarizes the response categories and binary agreement with the primary volumetric endpoint.

Metric	Value
Total evaluable patients	54
RANO progressive disease	8
RANO stable disease	31
RANO minor response	12
RANO partial response	3
RANO complete response	0
RANO tumor control (non-PD)	46/54 (85.2%)
Agreement with volumetric progression status	53/54 (98.1%)
Cohen kappa	0.930
Sensitivity of RANO PD for volumetric progression	88.9%
Specificity of RANO PD for volumetric progression	100%
Positive predictive value	100%
Negative predictive value	97.8%
Discordant case	P014: volumetric progression; RANO stable disease (+14.3% 2D product change)

Supplementary Table S6. Top 15 stable ΔT1C-WI features

Feature	Non-zero selections	Selection frequency	Mean coefficient	Mean absolute coefficient
delta_T1C__original|shape|LeastAxisLength	49	98%	0.716	0.716
delta_T1C__original|shape|MajorAxisLength	48	96%	0.859	0.859
delta_T1C__original|shape|MinorAxisLength	47	94%	0.742	0.742
delta_T1C__original|ngtdm|Strength	41	82%	-0.284	0.284
delta_T1C__original|glcm|MCC	40	80%	-0.345	0.345
delta_T1C__original|ngtdm|Coarseness	38	76%	-0.464	0.464
delta_T1C__original|ngtdm|Busyness	38	76%	0.321	0.321
delta_T1C__original|firstorder|Minimum	37	74%	-0.210	0.213
delta_T1C__original|shape|Maximum2DDiameterColumn	26	52%	0.391	0.391
delta_T1C__original|firstorder|10Percentile	23	46%	-0.207	0.209
delta_T1C__original|glcm|Imc1	21	42%	0.407	0.407
delta_T1C__original|glcm|Idmn	18	36%	-0.499	0.499
delta_T1C__original|shape|SurfaceArea	18	36%	0.221	0.221
delta_T1C__original|gldm|DependenceNonUniformity	18	36%	0.217	0.217
delta_T1C__original|gldm|SmallDependenceLowGrayLevelEmphasis	18	36%	0.139	0.152

Supplementary Figure S1. Exploratory elastic-net nomogram-style representation of the ΔT1C-WI model. Relative points are shown for the features retained in a descriptive full-cohort elastic-net refit, scaled according to the absolute magnitude of their coefficients. Positive and negative β values indicate the direction of association with 3-year volumetric progression. This figure is provided for descriptive interpretation only and was not used for performance estimation.

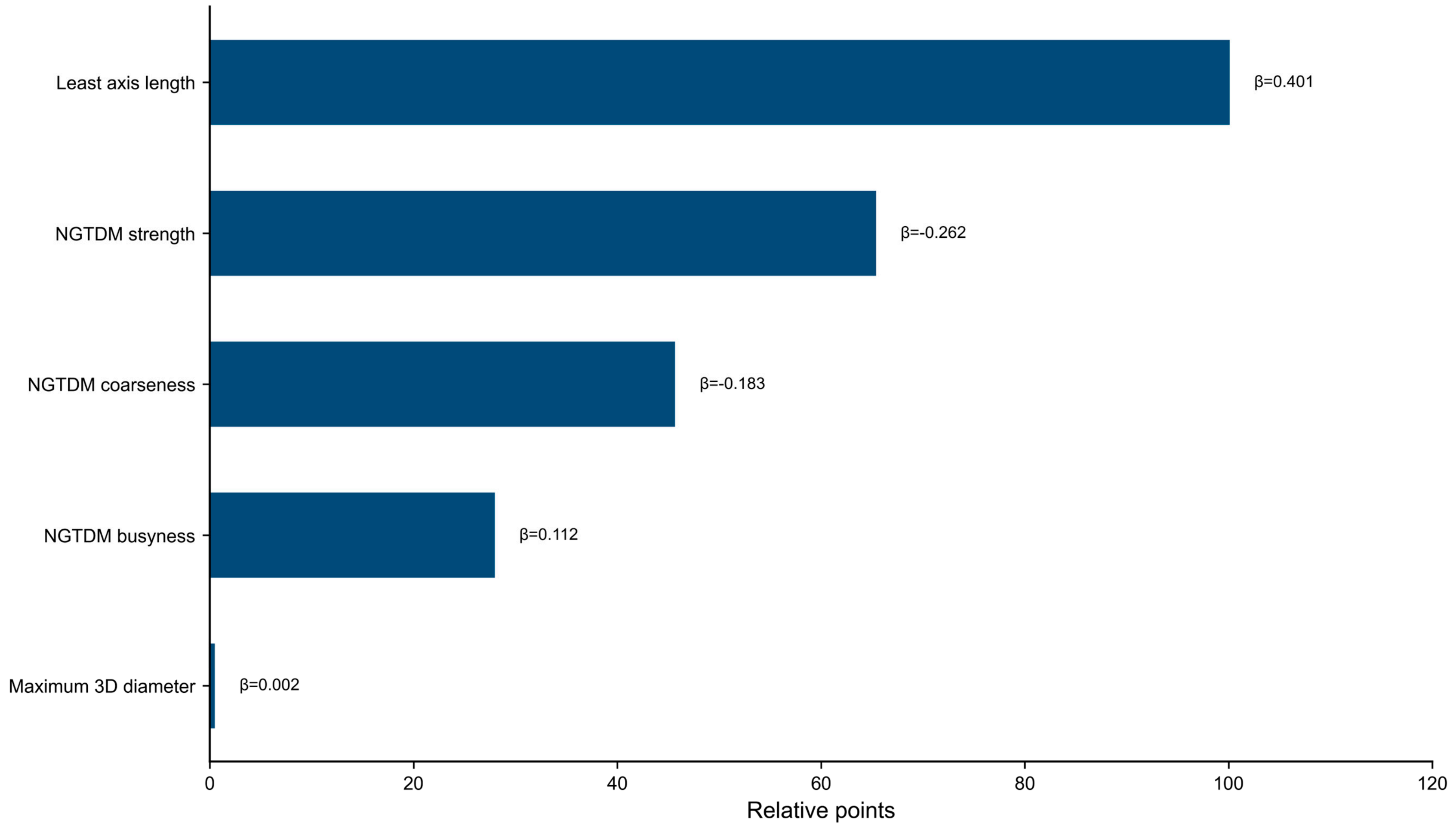

Supplementary data file

Supplementary Data S1. Anonymized extracted radiomic feature dataset, including pretreatment, 6-month, and delta features used in the analysis, together with the corresponding outcome labels (CSV).
